# Genome-wide characterization of aspartic protease (AP) gene family in *Populus trichocarpa* and identification of the potential PtAPs involved in wood formation

**DOI:** 10.1186/s12870-019-1865-0

**Published:** 2019-06-24

**Authors:** Shenquan Cao, Mengjie Guo, Chong Wang, Wenjing Xu, Tianyuan Shi, Guimin Tong, Cheng Zhen, Hao Cheng, Chuanping Yang, Nabil Ibrahim Elsheery, Yuxiang Cheng

**Affiliations:** 1 State Key Laboratory of Tree Genetics and Breeding, Northeast Forestry University, Harbin, Heilongjiang China; 2 Agriculture botany Department, Faculty of Agriculture, Tanta University, Tanta, Egypt

**Keywords:** *Populus trichocarpa*, Aspartic protease, Gene family, Promoter activity, Secondary cell wall, Glycosylation

## Abstract

**Background:**

Aspartic protease (AP) is one of four large proteolytic enzyme families that are involved in plant growth and development. Little is known about the AP gene family in tree species, although it has been characterized in *Arabidopsis*, rice and grape. The AP genes that are involved in tree wood formation remain to be determined.

**Results:**

A total of 67 AP genes were identified in *Populus trichocarpa* (PtAP) and classified into three categories (A, B and C). Chromosome mapping analysis revealed that two-thirds of the *PtAP* genes were located in genome duplication blocks, indicating the expansion of the AP family by segmental duplications in *Populus*. The microarray data from the *Populus* eFP browser demonstrated that *PtAP* genes had diversified tissue expression patterns. Semi-qRT-PCR analysis further determined that more than 10 *PtAPs* were highly or preferentially expressed in the developing xylem. When the involvement of the *PtAPs* in wood formation became the focus, many SCW-related *cis*-elements were found in the promoters of these *PtAPs*. Based on *PtAP*_*promoter*_*::GUS* techniques, the activities of *PtAP66* promoters were observed only in fiber cells, not in the vessels of stems as the xylem and leaf veins developed in the transgenic *Populus* tree, and strong GUS signals were detected in interfascicular fiber cells, roots, anthers and sepals of *PtAP17*_*promoter*_*::GUS* transgenic plants. Intensive GUS activities in various secondary tissues implied that *PtAP66* and *PtAP17* could function in wood formation. In addition, most of the PtAP proteins were predicted to contain N- and (or) O-glycosylation sites, and the integration of PNGase F digestion and western blotting revealed that the PtAP17 and PtAP66 proteins were N-glycosylated in *Populus*.

**Conclusions:**

Comprehensive characterization of the *PtAP* genes suggests their functional diversity during *Populus* growth and development. Our findings provide an overall understanding of the AP gene family in trees and establish a better foundation to further describe the roles of *PtAPs* in wood formation.

**Electronic supplementary material:**

The online version of this article (10.1186/s12870-019-1865-0) contains supplementary material, which is available to authorized users.

## Background

Aspartic proteases (APs; Enzyme Commission 3.4.23) are a group of proteolytic enzymes that exist widely in bacteria, fungi, animals and plants. They are a relatively simple class of enzymes that usually contain two aspartic acid residues within the conserved Asp-Thr/Ser-Gly motifs and are crucial for catalytic activity [1]. Generally, APs are most active under acidic pH (pH 2–6) and are specifically inhibited by pepstatin A. Unlike bacteria and fungi genomes with fewer family members [2–4], plant AP gene families are much larger. Fifty-one potential APs were found initially in Arabidopsis (*Arabidopsis thaliana*) and divided into three categories: typical APs, nucellin-like APs and atypical APs [5]. Subsequently, the Arabidopsis AP family was further enlarged to approximately 69 members [6]. Afterwards, 96 *OsAPs* and 50 *VvAPs* were identified in rice (*Oryza sativa* L*.*) and grape (*Vitis vinifera* L.) genomes, respectively [7, 8]. Although APs have been found in plants, knowledge of their functions is still lacking.

The first well-studied plant AP was phytepsin from barley (*Hordeum vulgare*), which possesses a plant-specific insert (PSI) domain and is localized in the vacuole; possibly, it plays a role in the active autolysis of tracheary elements and sieve cells [9, 10]. In the last decade, a limited number of studies indicated important roles for the APs in protein processing, signal transduction and stress responses [5, 11, 12]. Tobacco (*Nicotiana tabacum*) DNA-binding protease CND41 was involved in Rubisco turnover during leaf senescence [13]. OsAP65 was speculated to degrade a specific substrate and produce some substances necessary for pollen germination and pollen tube growth [14]. Arabidopsis nucellin-like aspartic protease APCB1 with a molecular cochaperone was revealed in the processing of Bcl-2-ASSOCIATED ATHANOGENE6 (BAG6) to trigger autophagy and defense resistance [15]. In Arabidopsis and rice, the extracellular AP (CONSTITUTIVE DISEASE RESISTANCE1, CDR1) mediated a peptide signal system involved in activation of inducible resistance mechanisms [16, 17]. In contrast, an apoplastic AP (APOPLASTIC EDS1-DEPENDENT1, AED1) suppressed systemic acquired resistance (SAR) by a feedback mechanism [18]. Another AP (ASPARTIC PROTEASE IN GUARD CELL1, ASPG1) participated in abscisic acid (ABA)-dependent responsiveness, and its overexpression could confer drought avoidance in Arabidopsis [19]. An additional report revealed the involvement of ASPG1 in Arabidopsis seed germination, which was associated with the degradation of seed storage proteins (SSPs) and regulation of gibberellic acid (GA) signaling [20].

In addition, APs play an important role in plant programmed cell death (PCD). In rice, *S5* participated in *indica-japonica* hybrid fertility and could stimulate endoplasmic reticulum (ER) stress, giving rise to PCD in the embryo sac [21, 22]. *AtMYB80* and rice *EAT1* controlled tapetum PCD by targeting the downstream aspartic protease genes *AtUNDEAD* and *OsAP25/OsAP37*, respectively. Silencing the expression of *AtUNDEAD* caused the premature tapetal PCD and resulted in pollen PCD [23]. Overexpressing *OsAP25* or *OsAP37* induced extensive cell death or premature death in the Arabidopsis tapetum [24]. Moreover, aspartyl protease PROMOTION OF CELL SURVIVAL1 (PCS1), as an anti-cell-death component, played crucial roles in embryonic development and reproduction processes and its loss-of-function mutation caused degeneration of gametophytes and excessive cell death in developing embryos [25]. In addition, two glycosylphosphatidylinositol (GPI)-anchored, aspartic protease *A36* and *A39* double mutants displayed unanticipated PCD in the pollen [26]. However, little is known about the AP gene family in tree species. The trees evolve many specific traits (such as large wood formation and perennial growth) that are different from herbaceous plants. Wood formation is complex process, with sequential events that include vascular cambium cell differentiation, cell elongation, secondary cell wall (SCW) thickening and PCD [27]. To date, the roles of plant APs in secondary cell wall formation or wood formation are not identified or reported in plants including the trees. In recent years, several AP proteins were recognized in plant cell wall proteomics [28]. In addition, cysteine proteases (CEP1, XCP1/2 and AtMC9) have been identified to function in SCW or PCD during secondary growth [29–31]. Hence, the AP genes that are involved in wood formation remain to be determined, and whether one to several *APs* might be as the indicative genes in the process of *Populus* wood formation arouses our interests.

Poplar is a fast-growing tree with high capacity for vegetative propagation, which has a large biomass accumulation in terrestrial ecosystem. They are extensively used for the pulp and paper industry, reforestation of lands and bioenergy feedstocks. As a model tree, *Populus trichocarpa* has attracted much attention, particularly by the availability of its genome sequences [32], which gave us an opportunity to carry out a comprehensive genome-wide analysis for exploring the potential functions of the AP genes in poplar. In the present study, we identified 89 *P. trichocarpa* AP genes, of which 67 *PtAPs* possess the complete ASP domain. Analysis of the phylogeny, gene organization, conserved motifs, gene duplications and expression patterns were carried out in this gene family. Based on SCW-related *cis*-elements and promoter::β-glucuronidase (GUS) expression analyses, some *PtAP* members were proposed to function in wood formation. Our study provides a significant foundation for further investigations into their potential roles in *Populus* growth and development and a better understanding of the AP gene family in a tree species.

## Results

### Genome-wide identification and phylogenetic analysis of *Populus* AP gene family

To identify the AP family members in *Populus*, systematic BLASTP analysis was performed using previously reported *Arabidopsis* AP protein sequences as the queries. The 92 nonredundant candidate sequences were identified in the *P. trichocarpa* genome (http://www.phytozome.net). Among them, 89 candidate sequences have the perfect open reading frame (ORF). Subsequently, these candidate AP genes were further confirmed by SMART and NCBI-CDD tests, and 67 genes were found to have the complete ASP domain (PF00026) with two conserved aspartic acid catalytic residues (Additional file 1 and Additional file 2). For further convenience, these 67 *Populus* AP genes were named *PtAP1* to *PtAP67* based on their order on the chromosomes (Additional file 1). The remaining 22 genes (named as *PtAP-Like1* to *22*) were eliminated in this study because they were partial or absent in the ASP domain through manual inspection (Additional file 3). The corresponding information for the *PtAP* genes, including gene symbol, locus, group, protein length (aa), molecular weight (MW), the predicted glycosylation sites and the proposed protein subcellular localization were shown in Additional file 1, Additional files 4 and 5.

**Fig. 1:**
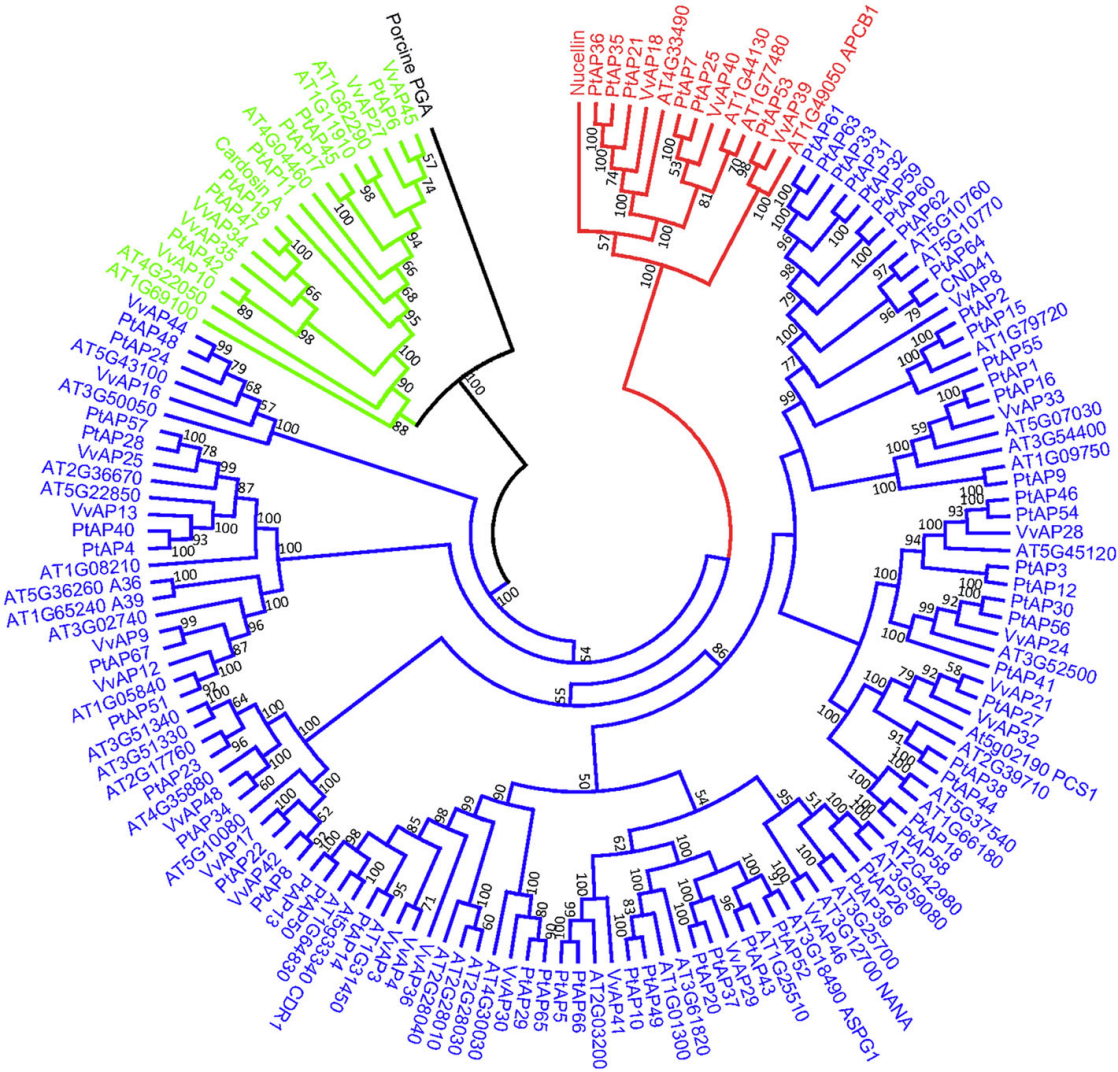
Phylogenetic analysis of *Populus,* grape and *Arabidopsis* AP protein sequences. Sixty-seven PtAPs, 30 VvAPs and 51 AtAPs were aligned with Clustal X 2.0, and the phylogenetic tree was constructed using the neighbor-joining method with 1000 bootstrap replication. Porcine pepsin A was used as the outgroup. Nucellin (U87148), CND41 (D26015) and cardosin A (CAB40134) were convenient to compare and define categories of the APs. All AP proteins were classified into three categories with different colors

To investigate the phylogenetic relationship, we constructed a phylogenetic tree with the 67 PtAPs, 30VvAPs and 51 AtAPs, as well as other known AP proteins (Nucellin, CND41 and cardosin A). The results indicate that these 67 PtAPs can be divided into three categories (A, B and C; Fig. 1), similar to those described in *Arabidopsis*, grape and rice [5, 7, 8]. Category A included 7 PtAPs and cardosin A. These proteins with the PSI domain (including SapB_1 and SapB_2) represent typical APs (Additional files 6 and 7), revealing more typical APs in *Populus* than those in *Arabidopsis.* In category B, 6 PtAPs consisted of nucellin-like APs, which contain two cysteines within the highly conserved sequences QCDYE and GCGYDQ [5]. The rest of the 54 PtAPs clustered into category C as atypical APs. Additionally, the dendrogram showed the closely related orthologous APs among *Populus*, grape and *Arabidopsis* (for instance, PtAP59–64/VvAP5–8/AT5G10760–10,770), suggesting that many ancestral AP genes existed prior to the divergence of *Populus*, grape and *Arabidopsis*.

### Gene structures and conserved motifs

**Fig. 2:**
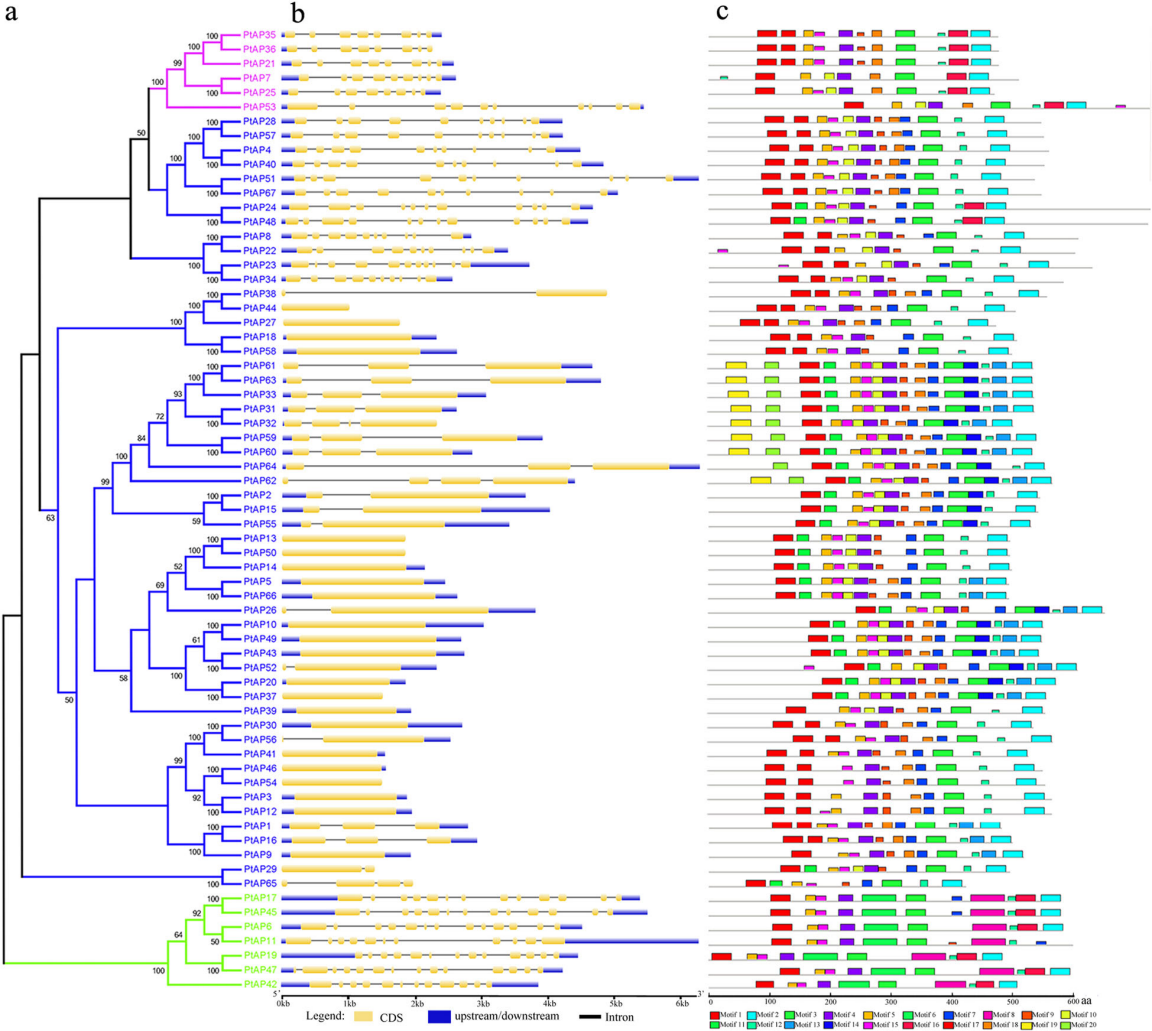
Phylogenetic tree, gene structures and protein motifs of 67 PtAPs. **a** The phylogenetic tree. Full-length protein sequences were aligned with Clustal X 2.0, and the phylogenetic tree was constructed using the neighbor-joining method. **b** Gene structure. Orange boxes represent exons, and black lines represent introns. The untranslated regions (UTRs) are indicated by blue boxes. The sizes of the exons and introns can be estimated using the scale at the bottom. **c** Protein motifs. Conserved motifs (1–20) are represented by different colored boxes while nonconserved sequences are shown by gray lines

In addition, we analyzed the exon/intron arrangements of the 67 *PtAP* genes based on their phylogenetic tree (Fig. 2a). The closest genes shared similar gene structures in terms of either the intron numbers or exon lengths, but *PtAP* genes in different categories exhibited different exon/intron structural features. Most genes from the category B had 8 exons except *PtAP53*, which contained 10 exons, similarly to a nearby subgroup. In the category C, the gene structures appeared to be more variable, with the exon numbers ranging from 1 to 12, but the number of exons was similar within each subgroup. Additionally, these results indicated a strong correlation between the phylogeny and exon/intron structures (Fig. 2b).

To gain insight into the specific regions of the PtAP proteins, we analyzed the distribution of conserved motifs, and 20 motifs were captured by the MEME online tool (Fig. 2c and Additional file 8). The length of the motifs ranged from 10 to 50 amino acids, and the number of conserved motifs varied from 9 to 15 in each of the PtAPs. Motifs 1, 2, 3, 4, 5, 12 and 15 appeared in nearly all members of PtAPs, whereas motifs 6 and 8 were specific to category A, and motifs 11, 13, 14 19 and 20 were found only in category C. Most of the PtAP proteins within each subgroup showed highly conserved motifs and different subgroups contained distinct conserved motifs.

### Chromosomal location and gene duplications

**Fig. 3:**
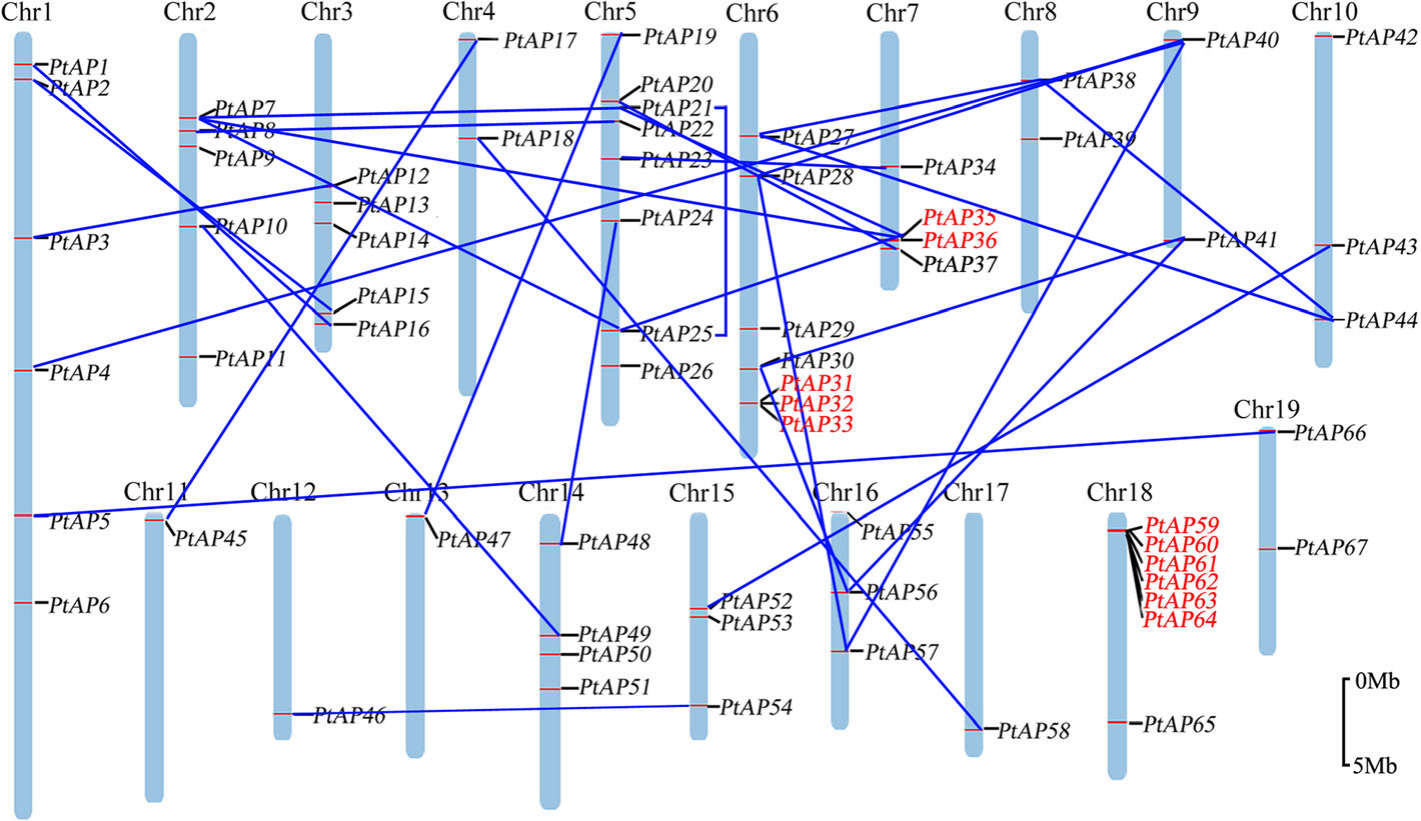
Chromosomal locations and duplicated gene pairs of 67 *PtAPs*. Each was mapped to the chromosome based on its physical location. The chromosome number (Chr01-Chr19) is indicated at the top. The segmental duplicated gene pairs were connecting with a solid blue line, and tandem duplicated genes were outlined with red color. The scale bar represents 5.0 Mb

We mapped 67 *PtAPs* on chromosomes by the PopGenIE v3 database (http://www.popgenie.org/). As shown in Fig. 3, the physical locations of these *PtAP*s on chromosomes were scattered and uneven. Chromosome 5 had the maximum number (eight members) of *PtAP* genes, followed by chromosomes 6 and 18, each with seven *PtAP* genes. Three *PtAP* genes were simultaneously distributed on chromosomes 10, 15, and 16, whereas chromosomes 11, 12, 13, and 17 had only one.

Segmental and tandem duplications are the main mechanisms leading to gene family expansion [33]. To elucidate the expanded mechanism of the *PtAP* gene family*,* we used duplicated blocks, which were established in a previous study [32], to determine the potential segmental duplications. As illustrated in Fig. 3, 30 gene duplication pairs corresponded to 42 *PtAP* genes. Among them, some subgroups of *PtAPs* were reciprocal duplication genes, such as *PtAP4*/*28*/*40*/*57*, *PtAP7*/*21*/*25*/*35*, *PtAP27*/*38*/*44* and *PtAP30*/*41*/*56*, implying that they might share a common ancestral gene. Moreover, *PtAP39*, *PtAP50* and *PtAP51* were duplicated on the corresponding blocks, but these duplicates (*Potri.010G130100*, *Potri.002G188800* and *PtAP-Like4*, respectively) were incomplete or had lost the ASP domain. In addition, approximately 18% (12 of 67) of the *PtAP* (*PtAP6*, *9*, *11*, *13*, *14*, *26*, *29*, *42*, *53*, *55*, *65*, and *67*) genes were located outside of any duplicated blocks. Generally, a gene cluster is the result of gene tandem duplication [34]. Here, we found that some *PtAP* genes were adjacent to each other (Fig. 3 and Additional files 1). For instance, *PtAP31* to *33*, *PtAP35*/*36*, and *PtAP59* to *64* were located sequentially in tandem on chromosomes 6, 7, and 18, respectively, implying that these genes might arise from recent tandem duplication events. These results indicate that both tandem and segmental duplications play a crucial role in the expansion of the *PtAP* gene family.

Additionally, we estimated the nonsynonymous substitution rate (Ka), the synonymous substitution rate (Ks) and the Ka/Ks ratio based on phylogenetic analysis and chromosomal distribution of *PtAPs*. The results showed that the approximate dates of the duplication events took place from 114.09 million years ago (Mya) to 9.47 Mya (Additional file 9). In view of the data showing divergence between *Populus* and *Arabidopsis* approximately 100 to 120 Mya, almost all the PtAP segmental duplications occurred after the *Populus* and *Arabidopsis* split. The Ka/Ks ratios of gene pairs were < 1.0, implying that these *PtAP* gene pairs were under the influence of purifying selection after duplication.

### Diversified expression patterns of PtAP genes

**Fig. 4:**
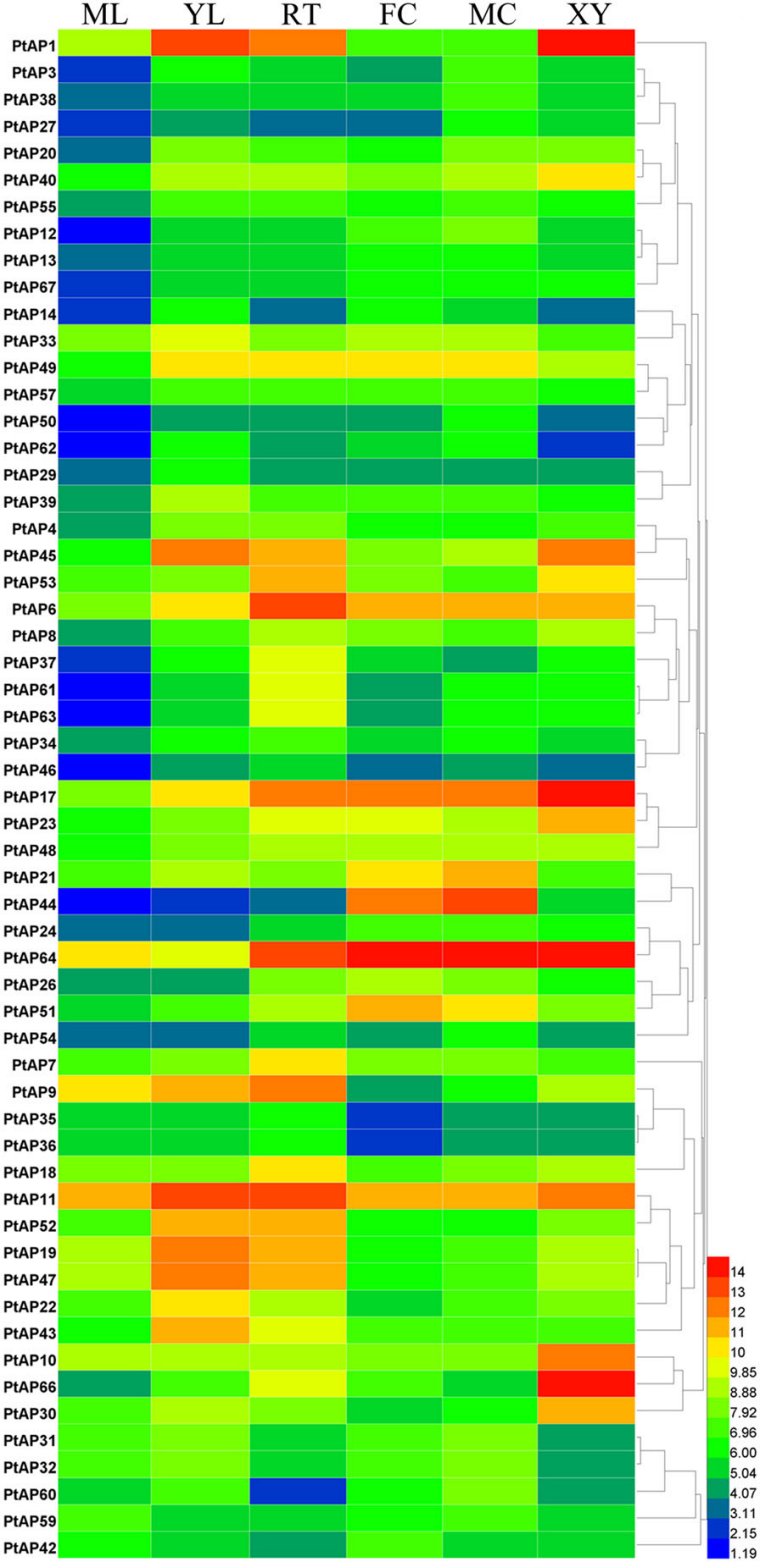
Hierarchical clustering of expression profiles of *PtAPs* in different tissues. The microarray data were downloaded from the Poplar eFP browser. Color scale at the right of the dendrogram represents log2 expression values. ML, mature leaf; YL, young leaf; RT, root; FC, female catkin; MC, male catkin; XY, xylem

To analyze the expression profiles of the *PtAP* genes, we reviewed their transcript abundance patterns across multiple tissues and organs (including root, developing xylem, young leaf, mature leaf, male catkin and female catkin) using data retrieved from the *Populus* eFP browser with corresponding probe sets (Additional file 10). In total, 57 *PtAP* genes were found in the microarray data (Additional file 11). The heat map demonstrated that most *PtAPs* had tissue-specific or preferential expression patterns (Fig. 4). *PtAP66* was specifically expressed in developing xylem; *PtAP17* and *PtAP64* were preferentially expressed in developing xylem, root and catkin; *PtAP1* and *45* were highly expressed in young leaf, root and developing xylem; *PtAP21*, *44* and *51* were expressed preferentially in catkins; and *PtAP6*/*11* and *PtAP19*/*47* showed higher expression level in root and young leaf, respectively. These data suggest that the *PtAPs* may be involved in multiple processes during *Populus* growth and development.

**Fig. 5:**
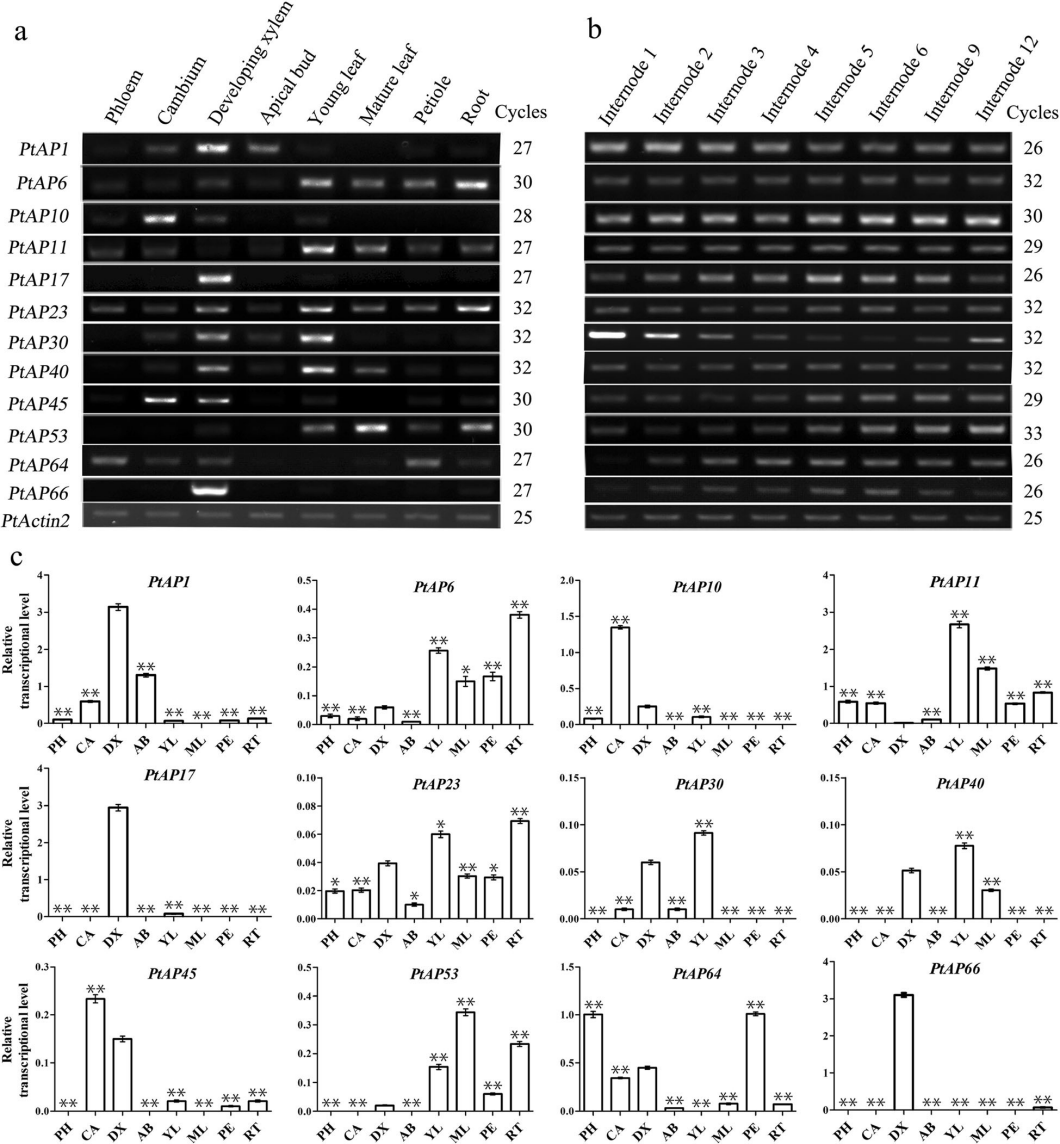
Semi-qRT-PCR and qRT-PCR analyses of 12 selected *PtAPs* in various tissues of *Populus trichocarpa*. **a-b** Expression of 12 selected *PtAP* genes using semi-qRT-PCR analysis. Different tissues included phloem (PH), cambium (CA), developing xylem (DX), apical bud (AB), young leaf (YL), mature leaf (ML), petiole (PE) and root (RT). Stem internodes included 1st to 6th, 9th and 12th internodes. **c** Expression of 12 selected *PtAP* genes by qRT-PCR analysis. The expression of *PtActin2* was used as an internal control. Data are means ± standard error of three technical replicate results. Statistically significant difference between DX and other tissues was determined by *t*-test. *P* < 0.05 was marked as * and *P* < 0.01 was marked as **

In addition, 12 *PtAP* genes with a higher eFP value (eFP > 1000) of developing xylem were selected to examine their expression levels by semi-qRT-PCR in phloem, cambium, developing xylem, apical bud, young leaf, mature leaf and petiole (Fig. 5a). Most *PtAP* gene expression profiles were consistent with the microarray data, whereas the expression levels of *PtAP30* and *PtAP40* in xylem were slightly lower than those in young leaves. This result may be due to an error caused by different standards of materials. In addition, the *PtAP1* was highly expressed in the apical bud, and *PtAP10* and *45* were preferentially expressed in the cambium (Fig. 5a), suggesting that they may function in these tissues. Furthermore, qRT-PCR analysis showed expression levels of 12 *PtAP* genes in different tissues (Fig. 5c), which is in agreement with the results from semi-qRT-PCR analysis.

We further explored the relationships of these *PtAP* genes that were highly expressed in xylem with the secondary cell wall formation. Since the 1st to 4th internodes (IN) showed primary growth with stem elongation, and the 5th IN below initiated the gradually lignified SCWs [35], we examined their expression levels in IN 1, 2, 3, 4, 5, 6, 9 and 12. As shown in Fig. 5b, the levels of *PtAP1* and *PtAP30* in the stem elongation region were dominant and tapered off as the stem secondary growth began. In contrast, *PtAP10*, *17*, *45*, *53, 64* and *66* showed the opposite trend in the expression levels, which correlated well with the switch to stem radial growth following activation of secondary growth. Our results indicate that these genes are likely to function in *Populus* secondary growth or wood formation.

### Analysis of the cis-elements related to SCW formation in the promoter region of the PtAPs

**Fig. 6:**
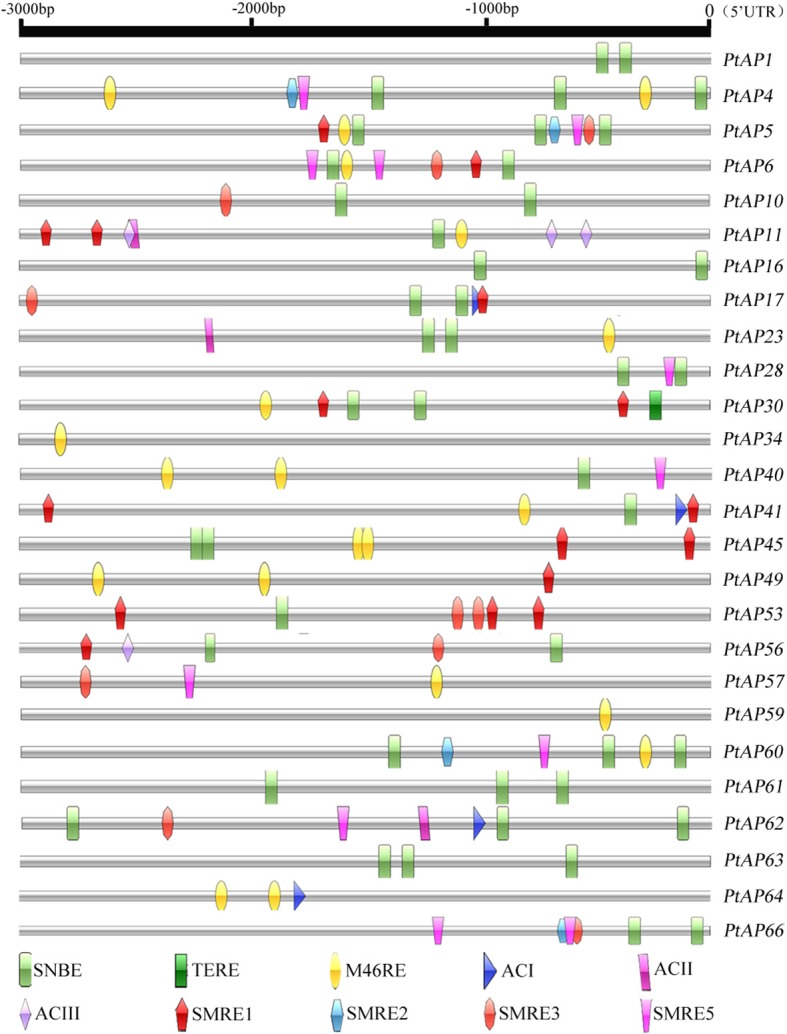
Analysis of secondary cell wall (SCW)-related *cis*-elements in promoters of 26 *PtAP* genes. The 3000 bp promoter regions (upstream DNA sequence of the 5′-UTR) of each *PtAP* gene were analyzed for SCW-related *cis*-elements. Different *cis*-elements were indicated by different colored diagrams

Transcriptional networks are responsible for the coordinated regulation of secondary wall biosynthetic gene expression during secondary wall formation. We presume that PtAPs may be in the downstream of transcriptional network regulating wood secondary wall formation and be targets of secondary wall-regulated transcription factors. In view of this, we analyzed the *cis*-elements related to SCW formation in the promoter regions of the 26 PtAPs (xylem eFP > 1000 and corresponding replication genes) with high or preferential expression levels in the developing xylem. As shown in Fig. 6, a total of 10 *cis*-elements were herein identified in these *PtAPs*. Most promoter regions (3000 bp) of these genes (except for *PtAP34, 49, 57, 59* and *64*) had *cis*-element SNBE. *PtAP4*, *5*, *6*, *11*, *23*, *30*, *34*, *40*, *41*, *45*, *49*, *57*, *59*, *60* and *64* contained *cis*-element M46RE. Nevertheless, *cis*-element TERE existed only in the promoter region of *PtAP30*. In addition, the promoter regions of *PtAP5*, *6*, *11*, *17*, *30*, *41*, *45*, *49*, *53* and *56* had the *cis*-elements SMRE1, and *PtAP17*, *41*, *62* and *64* contained the *cis*-elements ACI. To date, the SNBE, M46RE, TERE, SMRE and AC elements have been identified as being involved in SCW formation and (or) PCD during xylem development [36–41]. Therefore, our data indicated that these *PtAPs* should function in *Populus* wood formation.

### Promoter:GUS-based analysis of the SCW-related *PtAPs* in transgenic plants

**Fig. 7:**
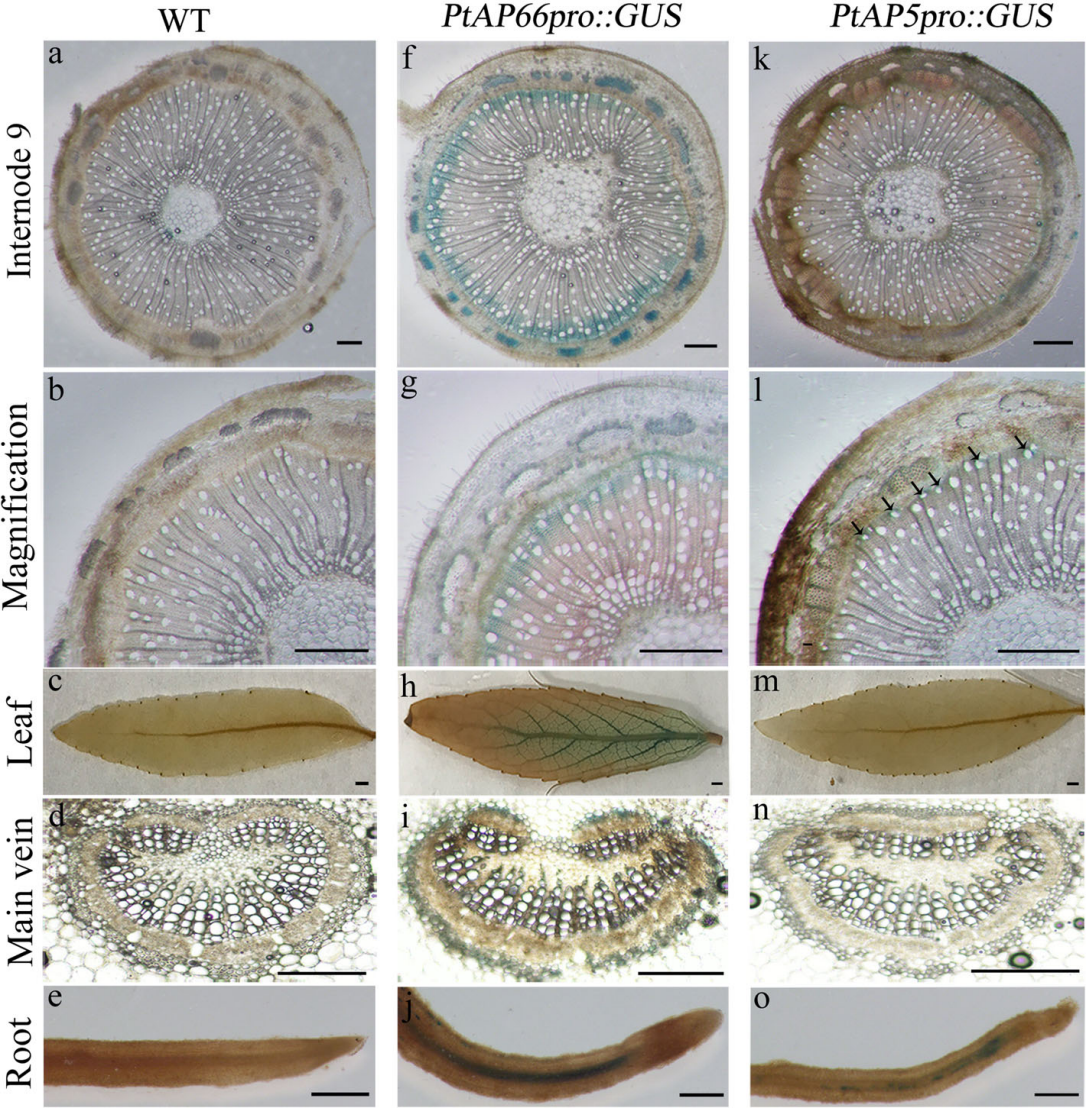
GUS assay of transgenic *Populus* driven by *PtAP66* and *PtAP5* promoters, respectively. **a**-**e** Wild type (WT) as a negative control; **f**-**j** *PtAP66promoter::GUS*; **k**-**o** *PtAP5promoter::GUS*. **a-b**, **f-g**, **k-l** Cross-sections of 9th stem internodes from 60-day-old WT, *PtAP66promoter::GUS* and *PtAP5promoter::GUS* young trees, respectively. **d-n** Cross-sections of leaf main veins from these young trees, respectively. Arrows indicate xylem vessels. Scale bars, 500 μm

**Fig. 8:**
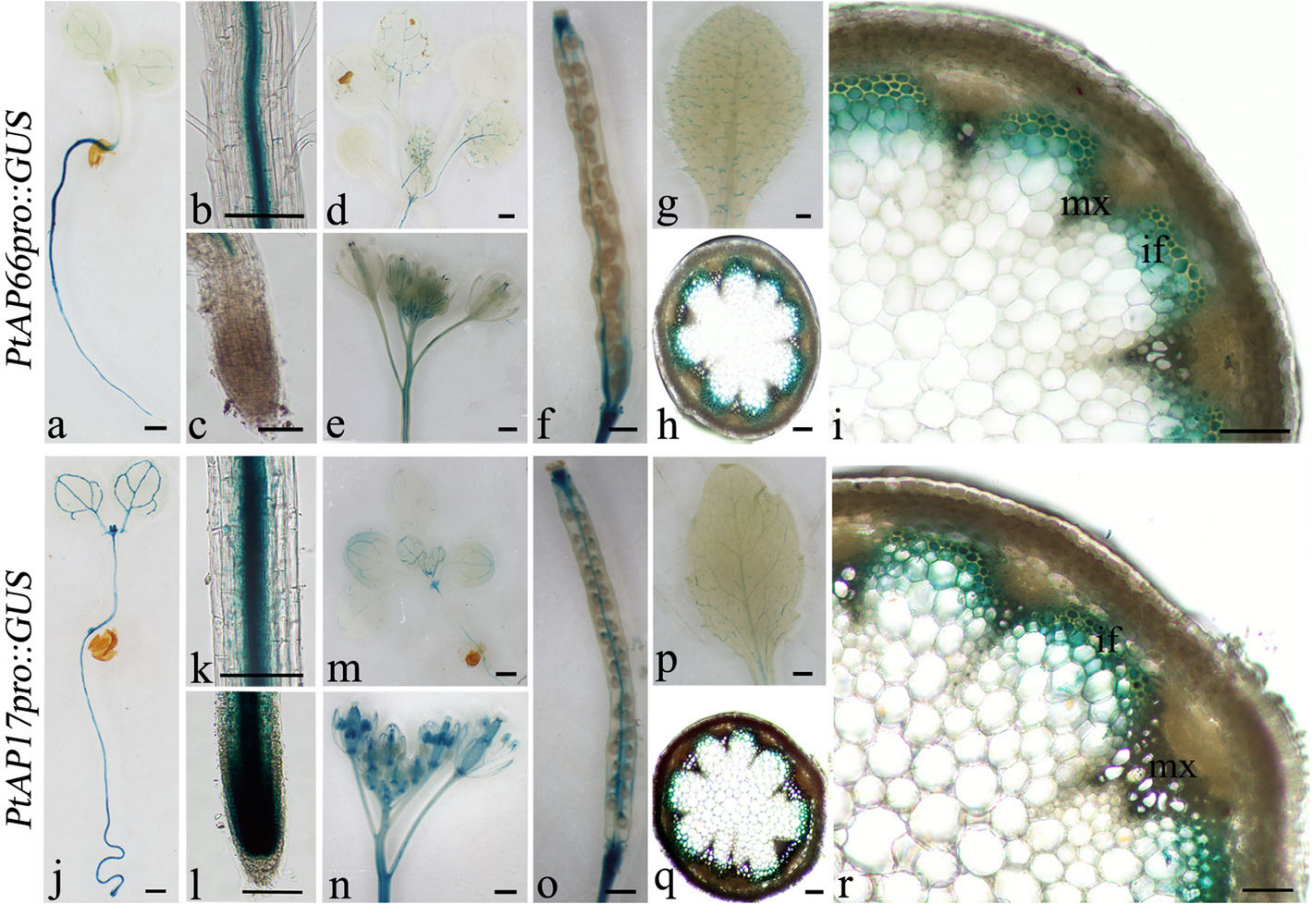
Activities of *PtAP66* and *PtAP17* promoters in transgenic *Arabidopsis*. GUS staining was detected in *PtAP66promoter::GUS* (**a**-**i**) and *PtAP17promoter::GUS* (**j**-**r**) transgenic plants. (**a**-**c**, **j**-**l**) 5-day-old seedlings, their roots and root tips, respectively. (**d**, **m**) Aerial parts of 2-week-old seedlings. (**e-h**, **n-q**) Inflorescences, siliques, rosette leaves and stem cross-sections of 40-day-old plants, respectively. (**i**, **r**) Magnification of stem cross-sections. if, interfascicular fiber; mx, metaxylem. Scale bars indicate 500 μm in **a**, **d**-**g**, **j** and **m**-**p** and 100 μm bars for **b**, **c**, **h**, **i**, **k**, **l**, **q** and **r**

Compared with other *PtAPs*, the *PtAP17* and *PtAP66* are most highly and specially expressed in the xylems (Fig. 5). In addition to this, PtAP17 and PtAP66 are predicted to be localized in vacuole and cell wall (Additional file 1), suggesting that they may be involved in different biological process during wood formation. Based on these, we firstly focused on the activities of *PtAP17* and *PtAP66* promoters using *PtAPpro::GUS* approach. In *PtAP66pro::GUS* lines, GUS activity was intensively detected in secondary phloem fibers, developing xylems, leaf main veins and root steles (Fig. 7f-j), which are the main parts of the secondary vascular system. Especially strong GUS signals were localized in the fibers, not in the vessels, of stems developing xylem and leaf main veins (Fig. 7f and i). To confirm the reliability of this, we further examined the activity of *PtAP66* promoter in transgenic *Arabidopsis*. As expected, GUS activities were still detected in main secondary vascular tissues (Fig. 8a-i), and the interfascicular fibers, not vessels in the metaxylem, displayed strong GUS activities (Fig. 8i). Interestingly, although *PtAP5pro::GUS* in transgenic *Populus* was moderately expressed on the level, a GUS signal could be observed in the vessels, but not in the fibers, of the developing xylems (Fig. 7k-l). *PtAP66* and *PtAP5*, a pair of segmental duplicated genes (Fig. 3), showed different expression patterns of their promoters in the developing xylems, suggesting the distinct roles of *PtAP66* and *PtAP5* in the wood formation. Additionally, we examined the activity of the *PtAP17* promoter in transgenic *Arabidopsis* plants (Fig. 8j-r). Intensive GUS signals were observed in stem interfascicular fibers, leaf main veins, and root steles of the *PtAP17pro::GUS* transgenic lines, as shown in the *PtAP66pro::GUS* transgenic lines. Moreover, GUS activities were detected in the root tip, anther and sepal of the *PtAP17pro::GUS* transgenic lines, which were distinct with the activities of the *PtAP66* promoter (Fig. 8c and e). These data also revealed that *PtAP17* should be involved in SCW formation.

### N-glycosylation analysis of the PtAP proteins

**Fig. 9:**
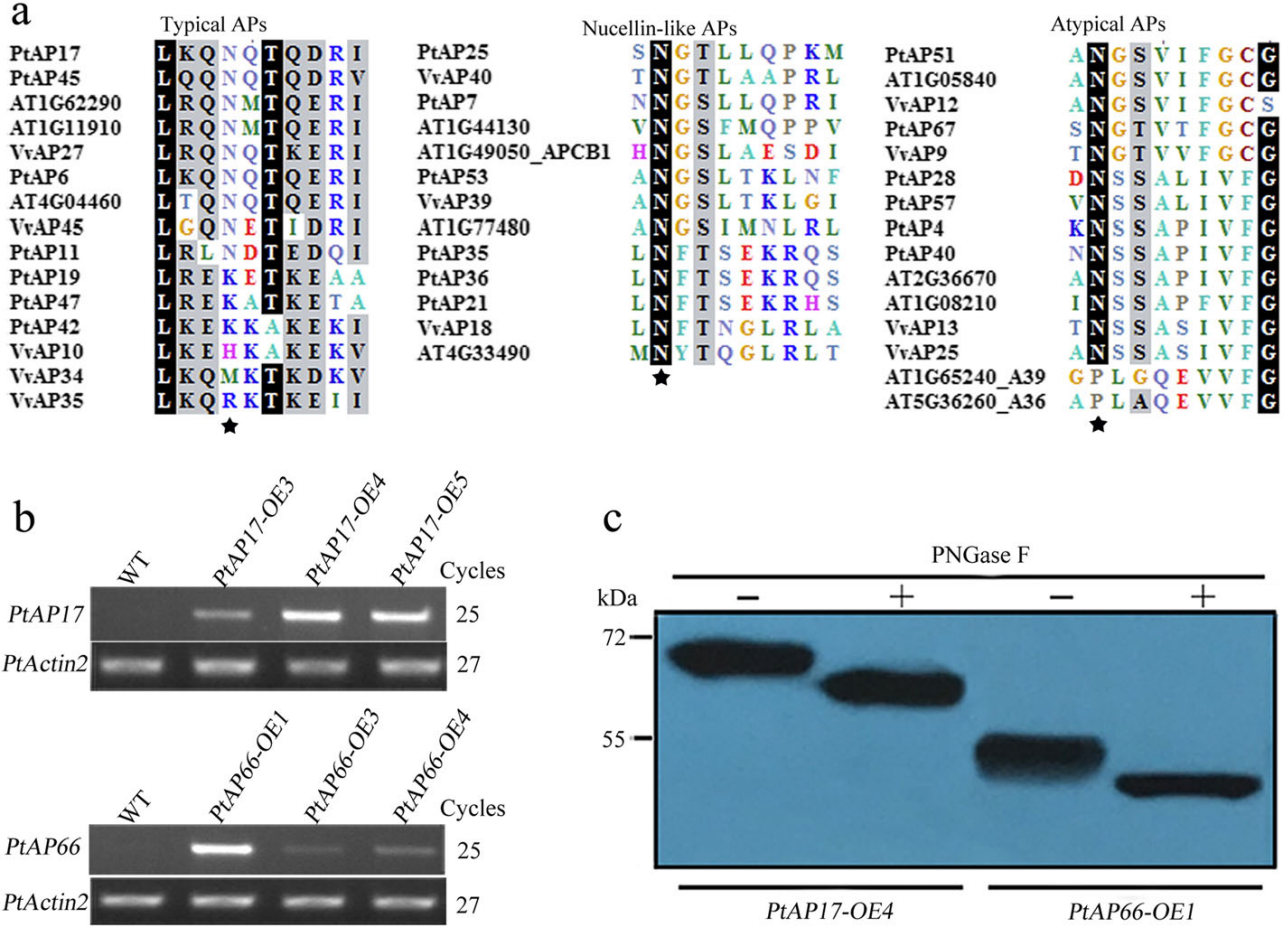
N-glycosylation analysis of PtAP proteins. **a** N-glycosylation site analysis of PtAP proteins from the categories A, B and C. The asterisk denotes the predicted N-glycosylation sites. Identical and conserved residues are shaded in black and gray, respectively. **b** Semi-qRT-PCR analysis of *PtAP17* and *PtAP66* expression in wild type (WT) and transgenic *Populus*. *PtActin2* was used as a loading control. **c** Protein extracts were isolated from the leaves of *PtAP17-OE4* and *PtAP66-OE1* transgenic *Populus*, respectively. After PNGase F treatments (with or without), protein extracts were separated using 10% SDS-PAGE and followed by immunoblotting with anti-FLAG antibody

The potential N- and O-glycosylation sites of the PtAP proteins were predicted as shown in Additional file 1 and Additional file 4. Except for PtAP4, 19 and 40, the 64 PtAP proteins had the N-glycosylation sites, and all PtAP members contained the O-glycosylation sites. We further examined if some predicted N-glycosylation sites within the crucial domains were conserved in plant APs (Fig. 9a; Additional file 4). All nucellin-like APs (category B) in *Populus*, grape and *Arabidopsis* contained the conserved N-glycosylation site between the invariant Tyr75 (pepsin numbering) and the GCGYDQ conserved sequences. Conversely, the N-glycosylation site in the PSI domain was not conserved in *Populus*, grape and *Arabidopsis* typical APs (category A). In category C, which had more members, the N-glycosylation site next to the invariant Tyr75 was incompletely conserved in the GPI-anchored AP subgroup, and the other AP subgroups had similar results.

To further verify the N-glycosylation of the PtAP proteins, we generated transgenic *Populus* overexpressing PtAP17-FLAG and PtAP66-FLAG (OE, fused with the FLAG tag at its carboxyl terminus), respectively (Fig. 9b). Western blot analysis using anti-FLAG antibody revealed the PtAP17- and PtAP66-FLAG proteins as being 67 kDa and 52 kDa, respectively, indicating these proteins are larger than their calculated MWs (Fig. 9c and Additional file 1). Digestion of their protein extractions with PNGase F resulted in faster protein mobility shifts of PtAP17- and PtAP66-FLAG on SDS-PAGE gels, indicating that PtAP17 and PtAP66 were N-glycosylated. Furthermore, their MWs after digestion of the PNGase F were still higher than those calculated (Fig. 9c and Additional file 1), suggesting the possible O-glycosylation of mature PtAP17 and PtAP66. These data provided the foundation for further investigations into the role of the glycosylation in the structure and function of PtAP proteins.

## Discussion

Increasing evidence has demonstrated that plant APs play important roles in protein processing, stress responses, PCD and reproduction [11]. To our knowledge, there is no report on involvement of plant APs in secondary cell wall formation or wood formation. Additionally, most studies on the APs mainly have focused on herbaceous plants, especially *Arabidopsis* and rice [15, 16, 19, 24–26, 42] and have neglected this family in tree species. In the present study, we performed a complete characterization of *PtAP* family in *Populus*, which included the phylogeny, gene organization, conserved motifs, gene duplications, expression patterns and glycosylation sites. Additionally, a set of *PtAPs* (*PtAP5*, *17*, *45* and *66*, etc.) were proposed to be involved in *Populus* wood formation, suggested by these data from tissue expression patterns, SCW-related *cis*-elements and *PtAP*_*promoter*_*::GUS* activities. These observations provide an overall understanding of the *AP* gene family in tree species and lay significant foundations for further investigation of their functions in tree growth and development.

In this study, we identified 67 AP genes in *P. trichocarpa* (Additional file 1), which were slightly more than those in *Arabidopsis* [6], although some gene duplication clusters were present. When we consider the ratio of 1.4~1.6 putative poplar homologs for the Arabidopsis gene [32], the number of *PtAP* gene family members cannot meet our expectations. The *Populus* genome has undergone at least three large-scaled genome-wide duplications [32], which might result in many partial and incomplete coding sequences, as noted in the 22 sequences eliminated from this study. Moreover, approximately 18% (12 of 67) of the *PtAP* genes were located outside of any duplicated blocks. Therefore, far fewer *PtAP* genes were observed in *P. trichocarpa* than we had expected. Even so, approximately 78% (52 of 67) of the *PtAP* genes were associated with segmental or tandem duplications, implying these genes existed as functional redundancies. For further selective pressure analysis, we found that purifying selection acted as a primary force during the AP gene family expansion in *Populus*, suggesting these duplicate genes might retain their ancestral functions [43].

PtAP family members within the same subgroup showed similar motifs and localization patterns (Fig. 2; Additional files 5 and 8). For example, the *PtAP4*, *28*, *40*, *51*, *57* and *67* were predicted to have a glycosylphosphatidylinositol (GPI) anchoring (Additional files 6 and 7), which is consistent with the A36 and A39 in *Arabidopsis* [26]. In addition, it is obvious that the same subgroup of *PtAP* genes shared similar exon/intron structures in three categories. In category C, 22 *PtAP*s lacked introns and 17 *PtAP*s lacked 5’UTRs (Fig. 2), which have been described in *Arabidopsis*, rice and grape [7, 8, 44]. Possibly, the common ancestral genes have already possessed this intron/exon structure. Although the introns do not contribute to protein sequences, their relative positions provide certain clues for use in predicting how genes and their corresponding proteins evolve and further contribute to the diversification of the gene family [45, 46]. Our data demonstrate the classification and evolutionary relationships of AP genes in *Populus*. This evidence also raised the possibility that plant APs in the same subgroup might have relatively conserved roles.

Gene expression patterns and function were highly correlated. PtAP family genes have diversified expression patterns, suggested by microarray data and semi-qRT-PCR analysis (Figs. 4 and 5). As the orthologous gene of *PCS1* that functions in embryonic development and reproduction processes, *PtAP44* was preferentially expressed in catkins [25]. Some other *PtAPs* (*AP17, 45, 64, 66*) have displayed higher transcript levels in xylems and positively correlated with stem lignified degrees. We sought these *PtAPs* in AspWood (http://aspwood.popgenie.org), which is a high spatial-resolution RNA sequencing database spanning secondary phloem, vascular cambium and wood tissues [47]. All of these *PtAP*s were highly expressed in the developing or lignified xylems (data not shown). Together, these expression data indicate that these *PtAP* genes should function in or associate with the SCW or PCD of wood formation. For this objective, we have filtered the SCW-related *cis*-elements in their promoter regions. All contained the SNBE or M46RE *cis*-elements (Fig. 6), which have been associated with SCW or PCD [36–39]. Furthermore, activities of *PtAP66* and *PtAP5* promoters in transgenic *Populus* (Fig. 7) suggest that they could be involved in wood formation. Interestingly, GUS staining data showed that *PtAP66* was expressed in fiber cells and not in vessels, but *PtAP5* was the opposite. This result is beyond our expectation because *PtAP66* and *PtAP5* are segmental duplication genes and have 87% amino acid sequence identities. The possibility is that the occurrence of mutations in their regulatory regions after gene duplication results in their different expression patterns. Subcellular localization prediction showed PtAP66 as a cell wall protein (Additional file 1). It is assumed that involvement of PtAP66 in SCW or wood formation possibly contributes to the degradation or the maturation of the proteins in the plasma membrane (PM) and/or cell wall. Presumably, these proteins may be structural wall proteins, wall modifying enzymes or PM-localized signaling proteins. Co-immunoprecipitation, yeast two-hybrid and proteomics approaches can help to seek the target proteins of PtAP66.

To date, only one type of protease, cysteine protease (XCP1/2 and AtMC9), has been demonstrated association with PCD and autolysis in vessels during SCW formation [29–31]. The understanding of PCD and autolysis in xylem fibers is limited. These PtAPs (PtAP6, 11, 17 and 45) in category A have been predicted in vacuoles and highly expressed in the lignified xylems (Figs. 4 and 5; Additional file 1), suggesting involvement of these PtAPs in PCD or autolysis during wood formation. Two SNBE *cis*-elements have been found in the promoter region of *PtAP17* (Fig. 6). An Arabidopsis orthologous gene (At4g04460) has been suggested as a downstream target of SECONDARY WALL-ASSOCIATED NAC DOMAIN PROTEIN1 (SND1) and VASCULAR-RELATED NAC-DOMAIN7 (VND7) from the microarray data, which was not tested [38]. However, *PtAP17* was specifically expressed in xylem fibers not vessels, as suggested by its promoter activity (Fig. 8), implying that the expression of *PtAP17* should be regulated by SND1 rather than VND7, which is a direct transcriptional regulator related to SCW and PCD in vessels [48]. These results suggested that the typical aspartic protease has functional differences in Poplar and Arabidopsis during SCW formation. Nevertheless, genetic evidence of this aspect needs to be obtained to identify its upstream transcription regulators.

Glycosylation, a common post-translational modification in eukaryotes, plays a crucial role in protein structure and function [49]. Most PtAP proteins had the potential N- and O-glycosylation sites (Additional file 1), and two N-glycosylated PtAPs (PtAP17 and 66) were confirmed by experimental data in this study (Fig. 9). Since most PtAPs have the signal peptides and are locate in secretory pathways (for instance, extracellular and vacuole), they need to be glycosylated. Whether some predicted N-glycosylation sites within the crucial domains are conserved was analyzed in each category of plant APs. Nucellin-like APs (category B) in *Populus*, grape and *Arabidopsis* have been found to contain the conserved N-glycosylation site between the invariant Tyr75 and the GCGYDQ motif, but plant APs of categories A and C have not (Fig. 9a). This suggests that different categories of plant AP proteins are diverse or confused in their employment of glycosylation modification. In previous studies, deglycosylated StAPs in *Solanum tuberosum* have affected subcellular accumulation in the apoplast and antifungal activity [50], and the replacement of the PSI domain of cardosin A (which has no glycosylated site) with the PSI domain of cardosin B (which has a glycosylated site) has changed the PSI mechanism of vacuolar sorting [51]. Presence or absence of the glycosylation sites in PtAP proteins could be a key structure in determining their molecular function or targeting route. In the future, the point-mutations to remove or add the glycosylation sites in PtAPs will uncover their roles in protein structure and molecular function.

Additionally, gain-of-functions of some plant APs would cause morphological changes. Misexpression of the *NANA* gene (*nana*) alters leaf morphology and causes a delayed flowering time [42]. Ectopic expression of *PCS1* causes a failure in anther dehiscence and male sterility [25]. Inducible expression *OsAP25* or *OsAP37* exhibits extensive cell death in *Arabidopsis* leaves and cotyledons [24]. Overexpression of the activation tagging mutant CDR1 causes dwarfing to virulent *Pseudomonas syringae* [16]. However, in overexpression of PtAP17 and PtAP66 transgenic *Populus*, we did not observe any differences on growth and development compared with the wild type. Whether the SCW and/or PCD during wood formation is mediated by PtAPs needs to be further investigated in these transgenic *Populus*. Next efforts to obtain these *PtAPs* knockout mutants will shed light on their genetic functions and the underlying molecular mechanisms.

## Conclusions

Here, we have presented an overall characterization of the AP family in *Populus*, including gene identification, gene structures, phylogenetic relationships, etc. Many *PtAP* genes have different tissue expression patterns, implying their functional diversity. Moreover, some *PtAP* genes (*PtAP5*, *17*, *45* and *66*, etc.) have been associated with *Populus* wood formation, most likely via the SCW and PCD processes, suggested by SCW-related *cis*-elements and *PtAP*_*promoter*_*::GUS* activity analyses. To date, the function of *AP* gene in plants including tree in SCW development or wood formation still remains to be determined. Therefore, we next investigate the phenotypes of the CRISPR/Cas9-targeted *PtAP* knockout *Populus* trees, which can clearly reveal the roles of these *PtAPs* in wood formation. In short, this study provides our understanding of the AP gene family in tree and provides a better foundation for determining the functional assignments of these genes.

## Methods

### Identification of AP genes in *P. trichocarpa*

A total of 51 AP protein sequences in *Arabidopsis* [5] were retrieved and used as queries to identify AP genes in the *P. trichocarpa* genome [52] using the BLASTP programs. Subsequently, these candidate sequences were analyzed with SMART database [53] and NCBI-CDD [54] tools to confirm that they have the complete Asp domains (PF00026) with two conserved aspartic acid catalytic residues. Partial and defective sequences were eliminated during manual inspection. The ExPASy program (https://web.expasy.org/compute_pi/) was used to calculate molecular weights of proteins. Five tools—Plant-mPLoc [55], LocTree3 [56], ProtComp 9.0 (http://linux1.softberry.com/), YLoc [57] and ngLOC [58]— were used to predict the protein subcellular locations, and the final results were presented according to the majority. N- and O-glycosylation sites were predicted in turn by NetNGlyc 1.0 (http://www.cbs.dtu.dk/services/NetNGlyc/) and NetOGlyc 4.0 server (http://www.cbs.dtu.dk/services/NetOGlyc/), with YES and NO representing positive and negative results, respectively.

### Multiple sequence alignment and phylogenetic analysis

Since 22 *Populus* candidates lacked a complete ASP domain, they were excluded from the phylogenetic analysis and further studies. The 67 PtAPs, 30VvAPs and 51 AtAPs (pepsin-like type), barley nucellins (GenBank accession no. U87148) [59], tobacco CND41 (D26015) [60], cardoon cardosin A (CAB40134) [61] and porcine pepsin A were aligned through Clustal X 2.0 [62]. The phylogenetic trees were constructed using MEGA 5.2 with the Neighbor-Joining (NJ) method under default parameters, and bootstrap analysis was conducted using 1000 replicates [63].

### Genomic organization and duplication analyses

The 67 *PtAP* genes were mapped on chromosomes by identifying their chromosomal locations according to the PopGenIE database [64]. Tandem duplication in the genome was defined as those closely related genes falling within 50 kb of one another [34]. Meanwhile, duplicated blocks were downloaded from the Plant Genome Duplication Database (PGDD) [65]. Genes were considered to have undergone segmental duplication if they were located on duplicated chromosomal segments. The number of Ka and Ks values was extracted from PGDD. The duplication time was estimated according to the formula: T = Ks/2λ. For *Populus*, the clock-like rate (λ) was 9.1 × 10^− 9^ [66].

### Gene and protein structure analysis

Both genomic sequences and corresponding coding sequences (CDS) of the *PtAPs* were loaded into the gene structure display server (GSDS) to identify the exon/intron arrangements [67]. The Multiple Expectation Maximization for Motif Elucidation (MEME) system was used to identify conserved motifs of AP protein [68]. Parameters were set as follows: the optimum motif width was set to 10–50, the maximum number of motifs to identify was set to 20 motifs, and all other parameters were defaulted. For each identified PtAP protein, putative signal peptide regions were identified using SignalP 4.1 (http://www.cbs.dtu.dk/services/SignalP/), ASP domains using CDD and SMART, and GPI-anchor addition sites using the big-PI Plant Predictor [69]. The diagram of protein structures was constructed with DOG 1.0 software [70].

### Secondary wall formation related *cis*-elements analyses

The 3 kb promoter sequences (upstream DNA sequence of the 5′-UTR) of *PtAP* genes (xylem eFP > 1000 and corresponding replication genes) were scanned for the SCW-related *cis*-elements by manual inspection. Here 10 *cis*-elements were found as listed follow: SNBE (WNNYBTNNNNNNNAMGNHW), TERE (CTTNAAAGCNA), M46RE (RKTWGGTR), ACI (ACCTACC), ACII (ACCAACC), ACIII (ACCTAAC), SMRE1 (ACCAAAT), SMRE2 (ACCAACT), SMRE3 (ACCAAAC) and SMRE5 (ACCTAAT). The diagrams of these *cis-*elements in promoter regions were drawn using the DOG 1.0 software.

### Plant materials and growth conditions

Wild type *Populus trichocarpa* (Nisqually-1) was planted in the greenhouse of Northeast Forestry University under conditions with a long day (16 h light/ 8 h dark) at 23–25 °C. More than 9 young trees (100-days old) were pooled for detecting the expression levels in different tissues and organs, and three biological replications were performed. Samples included apical bud, phloem, cambium, developing xylem, young leaf, mature leaf, petiole and root. The 1th to 6th, 9th and 12th internodes, developing xylem, cambium and phloem samples were collected as described in a previous study [71].

Arabidopsis (*Arabidopsis thaliana*; ecotype Columbia) plants were grown in the greenhouse (16 h of light/ 8 h of dark) at a light intensity of 120 μmol photons m^− 2^ s^− 1^ at 22 °C. The 5 and 14-day-old seedlings for GUS staining were grown on sterilized 1/2 Murashige and Skoog plates supplemented with 1% sucrose.

### Microarray data analysis, RNA isolation, semi-qRT-PCR and qRT-PCR analyses

Tissue-specific expression data on the *PtAPs* were downloaded from the *Populus* eFP browser [72]. Probe sets corresponding to *PtAP* genes were identified using an online Probe Match tool available at NetAffx Analysis Center (http://www.affymetrix.com/). The heat map was generated by Heat map illustrator (HemI) with the default settings [73].

Total RNA was isolated with plant RNA extraction reagents (Bio-Flux, China). For each sample, 1 μg of total RNA was reverse-transcribed into total cDNAs using the PrimeScript RT reagent Kit (TaKaRa, China). The expression of the *PtActin2* was used as an internal control of the same total cDNAs. Expression of the *PtAPs* in different tissues and internodes were examined by semi-qRT-PCR using Ex*Taq* (TaKaRa, China). The reaction mixture (20 μl) consisted of 1 μl of cDNA template, 0.5 μl of each gene-specific primer (10 μM), 2 μl 10× Ex*Taq* buffer (Mg^2+^ free), 2 μl dNTP mixture (2.5 mM each), 1.6 μl MgCl_2_ and 0.1 μl Ex*-Taq*. The PCR parameters were 94 °C for 3 min; followed by 25–32 cycles of 94 °C for 30 s, 58 °C for 30 s, 72 °C for 30 s; and a final step at 72 °C for 7 min. The resulting PCR products were run on a 2% agarose gel and visualized under a UV transilluminator to verify product sizes. All primers used in the present study for semi-qRT-PCR are listed in Additional file 12. Each reaction was conducted in triplicate to ensure the reproducibility of the results.

To validate the *PtAP* gene expression profiles, qRT-PCR analysis of these genes were performed with the same cDNA templates and primers that were used in semi-qRT-PCR experiment above. The qRT-PCR experiments were performed with SYBR Green (TaKaRa, China) in the ABI Prism 7500 system (Applied Biosystems, USA) according to the manufacturer’s instructions. The reaction mixture (20 μl) consisted of 10 μl 2 × TB Green *Premix Ex Taq* II (Tli RNaseH Plus), 0.8 μl of each gene-specific primer, 0.4 μl ROX Reference Dye II, 1 μl cDNA template and 7 μl distilled deionized H_2_O. The PCR parameters as follows: 95 °C for 30 s; 40 cycles of 95 °C for 5 s, 60 °C for 15 s, 72 °C for 30 s. *PtActin2* was used as an internal control and the comparative Ct (2^-△Ct^) method was used to calculate gene expression levels. Three technical replicates were done for each sample.

### Plasmid construction and *Agrobacterium*-mediated transformation

Genomic DNA was extracted from leaves using a plant genomic DNA extraction kit (Bioteke, China). With this genomic DNA being used as the template, approximately 3 kb promoter regions of *PtAP17* and *PtAP66* were amplified by PCR and cloned into pENTR/D-TOPO (Gateway, Invitrogen) to generate entry clones (Additional file 12). After DNA sequencing, the promoter fragments from entry clones were constructed into a Gateway binary vector pGWB3 by the LR recombination reaction. The constructs were transformed into *Agrobacterium tumefaciens* strain GV3101 and introduced into the *Arabidopsis* plants using the floral-dip method [74].

The CDS of *PtAP17* and *PtAP66* from Phytozome was amplified using the xylem cDNA as a template to construct overexpression vectors of pGWB11-PtAP17/PtAP66-FLAG, and the primers are listed in Additional file 12. After determination of DNA sequencing, the resultant constructs (overexpression and promoter) were introduced into *A. tumefaciens* strain GV3101 for *Populus* transformation as described previously [75].

### GUS staining

For each transgenic plant, at least six independent lines were analyzed for GUS staining. Consistent results from GUS staining in representative lines were recorded and wild-type plants negative to GUS histochemical assay were used as the controls. GUS staining was carried out as described [76]. After the GUS signal was developed, the chlorophylls of samples were cleared using 75% (v/v) ethanol several times. The images for freehand sections of *Arabidopsis* stems and *Populus* veins were captured by a BX43 stereomicroscope (Olympus, Japan) and the other tissues were captured by SZX7 stereomicroscope (Olympus, Japan).

### Deglycosylation by PNGase F treatment and western blot

Transgenic plant materials were ground in liquid nitrogen and homogenized in protein extraction buffer (50 mM Tris-HCl, 200 mM NaCl, 2% SDS, 5 mM DTT, pH 8.0). The suspensions were centrifuged at 18,000 xg for 5 min and the supernatants (protein extracts) were used for protein deglycosylation with/without PNGase F (New England Biolabs, UK). These treated protein extracts were then resolved in 10% SDS-PAGE gel and transferred into PVDF membranes. Western blotting was performed using anti-FLAG antibody (Abmart, China) and Pierce ECL chemiluminescent Substrate (Thermo, USA).

### Statistical analysis

Student’s *t*-test was performed to determine significant difference on RT-qPCR gene expression data using statistical software SPSS19.0 (Chicago, USA). Statistically significant differences at different levels (*P* < 0.05 and *P* < 0.01) were marked as *and **, respectively.

## Additional files

Additional file 1: **Table S1.** AP gene family in *Populus trichocarpa. (DOCX 24 kb)*

Additional file 2: **Figure S1.** Multiple sequence alignment of ASP domains in 67 *PtAPs*. The alignment was generated using Clustal X program with manual modification. The two conserved catalytic regions (DT/SG) of ASP domains and the invariant Tyr75 (pepsin numbering) are indicated. Identical and conserved residues are shaded in black and gray, respectively. (JPG 13825 kb)

Additional file 3: **Table S2.** The 22 *PtAP-like* genes eliminated in this study. (XLSX 14 kb)

Additional file 4: **Table S3.** Prediction analysis of N- and O-glycosylation sites in the 67 PtAPs. (XLSX 43 kb)

Additional file 5: **Table S4.** Prediction analysis of subcellular localization of the 67 PtAPs. (XLSX 36 kb)

Additional file 6: **Figure S2.** Signal peptides and conserved domains of 67 PtAPs. Phylogenetic analysis of 67 PtAPs in the left. The distribution of signal peptides and conserved domains of PtAPs. (JPG 1762 kb)

Additional file 7: **Table S5.** Backbones of 67 PtAP protein precursors. (DOCX 41 kb)

Additional file 8: **Figure S3.** Conserved motifs (1–20) identified by MEME online tool in 67 PtAPs. (JPG 1685 kb)

Additional file 9: **Table S6.** Estimated divergence period of PtAP gene pairs in *Populus trichocarpa. (DOCX 15 kb)*

Additional file 10: **Table S7.** Probes of *PtAP* genes in *Populus trichocarpa. (DOCX 32 kb)*

Additional file 11: **Table S8.** The eFP values of 57 *PtAPs* in six tissues downloaded from the Poplar eFP browser. (XLSX 13 kb)

Additional file 12 **Table S9.** All primers used in this study. (XLSX 10 kb)

## Data Availability

The datasets supporting the conclusions of this article are included within the article and its Additional files.

## Abbreviations

AP: Aspartic protease
BLAST: Basic local alignment search tool
CDS: Coding sequence
CRISPR/Cas9: Clustered regularly interspaced short palindromic repeats/CRISPR-associated protein-9 nuclease
eFP: Electronic fluorescent Pictograph
GPI: Glycosylphosphatidylinositol
GUS: β-glucuronidase
MW: Molecular weight
ORF: Open reading frame
PCD: Programmed cell death
PtAP: *Populus trichocarpa* AP
qRT-PCR: Quantitative reverse transcription polymerase chain reaction
SCW: Secondary cell wall
SDS-PAGE: Sodium dodecyl sulfate-polyacrylamide gel electrophoresis
Semi-qRT-PCR: Semi-quantitative reverse transcription polymerase chain reaction
SND1: Secondary wall-associated NAC domain protein 1
UTR: Untranslated region
VND7: Vascular-related NAC-domain 7
