# Supplementary material for: Genome-wide characterization of aspartic protease (AP) gene family in Populus trichocarpa and identification of the potential PtAPs involved in wood formation
Source: BMC Plant Biol. 2019 Jun 24;19:276. doi: 10.1186/s12870-019-1865-0 (PMC6591973; doi:10.1186/s12870-019-1865-0)
Supplement: Supplementary file 1 — Table S1. AP gene family in Populus trichocarpa. (DOCX 24 kb) [file 12870_2019_1865_MOESM1_ESM.docx]

**Table S1 AP gene family in *Populus trichocarpa***

| **Gene symbol** | **Gene locus** | **Group** | **Protein length (aa)** | **Molecular weight (MW)** | **N-/O-glycosylation sites^(a)^** | **Subcellular**  **Localization^(b)^** |
| --- | --- | --- | --- | --- | --- | --- |
| PtAP1 | Potri.001G028200 | C | 425 | 45612.45 | YES/YES | Ext |
| PtAP2 | Potri.001G041700 | C | 482 | 52451.86 | YES/YES | Ext |
| PtAP3 | Potri.001G158600 | C | 496 | 54593.76 | YES/YES | Not clear |
| PtAP4 | Potri.001G213600 | C | 506 | 54313.82 | NO/YES | PM, Ext |
| PtAP5 | Potri.001G306200 | C | 439 | 46992.62 | YES/YES | Not clear |
| PtAP6 | Potri.001G356900 | A | 515 | 56167.93 | YES/YES | Vac |
| PtAP7 | Potri.002G081400 | B | 460 | 50592.11 | YES/YES | Not clear |
| PtAP8 | Potri.002G092100 | C | 536 | 59094.05 | YES/YES | PM |
| PtAP9 | Potri.002G104600 | C | 458 | 48712.68 | YES/YES | Not clear |
| PtAP10 | Potri.002G171700 | C | 490 | 52364.23 | YES/YES | ER |
| PtAP11 | Potri.002G228300 | A | 530 | 58007.21 | YES/YES | Vac |
| PtAP12 | Potri.003G076300 | C | 496 | 54924.34 | YES/YES | Not clear |
| PtAP13 | Potri.003G087900 | C | 440 | 47419.47 | YES/YES | Ext |
| PtAP14 | Potri.003G105300 | C | 443 | 48275.77 | YES/YES | Ext |
| PtAP15 | Potri.003G185100 | C | 481 | 52866.53 | YES/YES | Ext |
| PtAP16 | Potri.003G195500 | C | 441 | 47406.86 | YES/YES | Not clear |
| PtAP17 | Potri.004G007600 | A | 512 | 55337.22 | YES/YES | Vac |
| PtAP18 | Potri.004G085000 | C | 446 | 48878.09 | YES/YES | Not clear |
| PtAP19 | Potri.005G002800 | A | 427 | 46461.96 | NO/YES | Vac |
| PtAP20 | Potri.005G063000 | C | 502 | 53828.04 | YES/YES | ER |
| PtAP21 | Potri.005G069600 | B | 430 | 47274.79 | YES/YES | Ext |
| PtAP22 | Potri.005G079900 | C | 531 | 57895.66 | YES/YES | PM, Vac |
| PtAP23 | Potri.005G108700 | C | 556 | 60392.33 | YES/YES | PM |
| PtAP24 | Potri.005G144600 | C | 641 | 70820.49 | YES/YES | PM, Ext |
| PtAP25 | Potri.005G179900 | B | 424 | 46555.27 | YES/YES | Ext |
| PtAP26 | Potri.005G204600 | C | 579 | 64376.42 | YES/YES | Ext |
| PtAP27 | Potri.006G087600 | C | 416 | 45239.41 | YES/YES | PM, ER |
| PtAP28 | Potri.006G118800 | C | 494 | 53069.61 | YES/YES | PM, Ext |
| PtAP29 | Potri.006G179500 | C | 438 | 49057.68 | YES/YES | Ext |
| PtAP30 | Potri.006G204700 | C | 469 | 51044.37 | YES/YES | Ext |
| PtAP31 | Potri.006G232400 | C | 474 | 50876.52 | YES/YES | Ext |
| PtAP32 | Potri.006G232500 | C | 443 | 47250.64 | YES/YES | Not clear |
| PtAP33 | Potri.006G232600 | C | 473 | 50844.74 | YES/YES | ER |
| PtAP34 | Potri.007G063800 | C | 514 | 56858.46 | YES/YES | PM |
| PtAP35 | Potri.007G099200 | B | 429 | 46753.30 | YES/YES | PM |
| PtAP36 | Potri.007G099300 | B | 430 | 47058.84 | YES/YES | PM |
| PtAP37 | Potri.007G106300 | C | 488 | 52637.43 | YES/YES | ER, Chl |
| PtAP38 | Potri.008G058000 | C | 490 | 54581.05 | YES/YES | ER |
| PtAP39 | Potri.008G115900 | C | 486 | 53123.68 | YES/YES | Not clear |
| PtAP40 | Potri.009G001700 | C | 499 | 53183.55 | NO/YES | Ext |
| PtAP41 | Potri.009G162400 | C | 462 | 50921.02 | YES/YES | Ext |
| PtAP42 | Potri.010G003400 | A | 495 | 54483.49 | YES/YES | Vac |
| PtAP43 | Potri.010G128200 | C | 484 | 51980.17 | YES/YES | ER |
| PtAP44 | Potri.010G201400 | C | 444 | 49404.87 | YES/YES | ER |
| PtAP45 | Potri.011G007600 | A | 512 | 55164.95 | YES/YES | Vac |
| PtAP46 | Potri.012G118000 | C | 483 | 52801.26 | YES/YES | Ext |
| PtAP47 | Potri.013G002200 | A | 526 | 57307.52 | YES/YES | Vac |
| PtAP48 | Potri.014G020200 | C | 637 | 70843.51 | YES/YES | PM, Ext |
| PtAP49 | Potri.014G099400 | C | 488 | 52179.14 | YES/YES | ER |
| PtAP50 | Potri.014G114400 | C | 440 | 46891.85 | YES/YES | Ext |
| PtAP51 | Potri.014G146400 | C | 485 | 53307.00 | YES/YES | PM |
| PtAP52 | Potri.015G051800 | C | 533 | 57588.64 | YES/YES | PM, ER |
| PtAP53 | Potri.015G053300 | B | 655 | 72795.29 | YES/YES | Ext |
| PtAP54 | Potri.015G113100 | C | 486 | 52430.60 | YES/YES | Ext |
| PtAP55 | Potri.016G000600 | C | 471 | 50145.46 | YES/YES | PM, Ext |
| PtAP56 | Potri.016G071900 | C | 497 | 54355.26 | YES/YES | Ext |
| PtAP57 | Potri.016G096700 | C | 498 | 53751.86 | YES/YES | PM |
| PtAP58 | Potri.017G131800 | C | 441 | 48526.48 | YES/YES | Not clear |
| PtAP59 | Potri.018G014500 | C | 477 | 51077.45 | YES/YES | PM, Ext |
| PtAP60 | Potri.018G014600 | C | 471 | 49961.61 | YES/YES | PM, Ext |
| PtAP61 | Potri.018G014700 | C | 472 | 50442.10 | YES/YES | Ext |
| PtAP62 | Potri.018G014800 | C | 500 | 54519.64 | YES/YES | Ext |
| PtAP63 | Potri.018G014900 | C | 472 | 50442.14 | YES/YES | Ext |
| PtAP64 | Potri.018G015100 | C | 490 | 51487.09 | YES/YES | PM |
| PtAP65 | Potri.018G106500 | C | 374 | 41562.35 | YES/YES | Ext |
| PtAP66 | Potri.019G002100 | C | 439 | 47083.44 | YES/YES | Ext |
| PtAP67 | Potri.019G054900 | C | 483 | 52476.54 | YES/YES | PM |

***^a^*** Protein N- and O-glycosylation sites were predicted by NetNGlyc 1.0 and NetOGlyc 4.0 server, respectively. “YES” represented at least exist one glycosylation sites (positive) and “NO” represented negative results.

***^b^*** Protein subcellular localization was predicted by softwares (Plant-mPLoc, LocTree3, ProtComp 9.0, Yloc, and ngLOC), and the final results were presented according to the majority, otherwise not clear. Chl, chloroplast; ER, endoplasmic reticulum; Ext, extracellular; PM, plasma membrane; Vac, vacuole.
