# Supplementary figures and images for: Genome-wide characterization of aspartic protease (AP) gene family in Populus trichocarpa and identification of the potential PtAPs involved in wood formation
Source: BMC Plant Biol. 2019 Jun 24;19:276. doi: 10.1186/s12870-019-1865-0 (PMC6591973; doi:10.1186/s12870-019-1865-0)

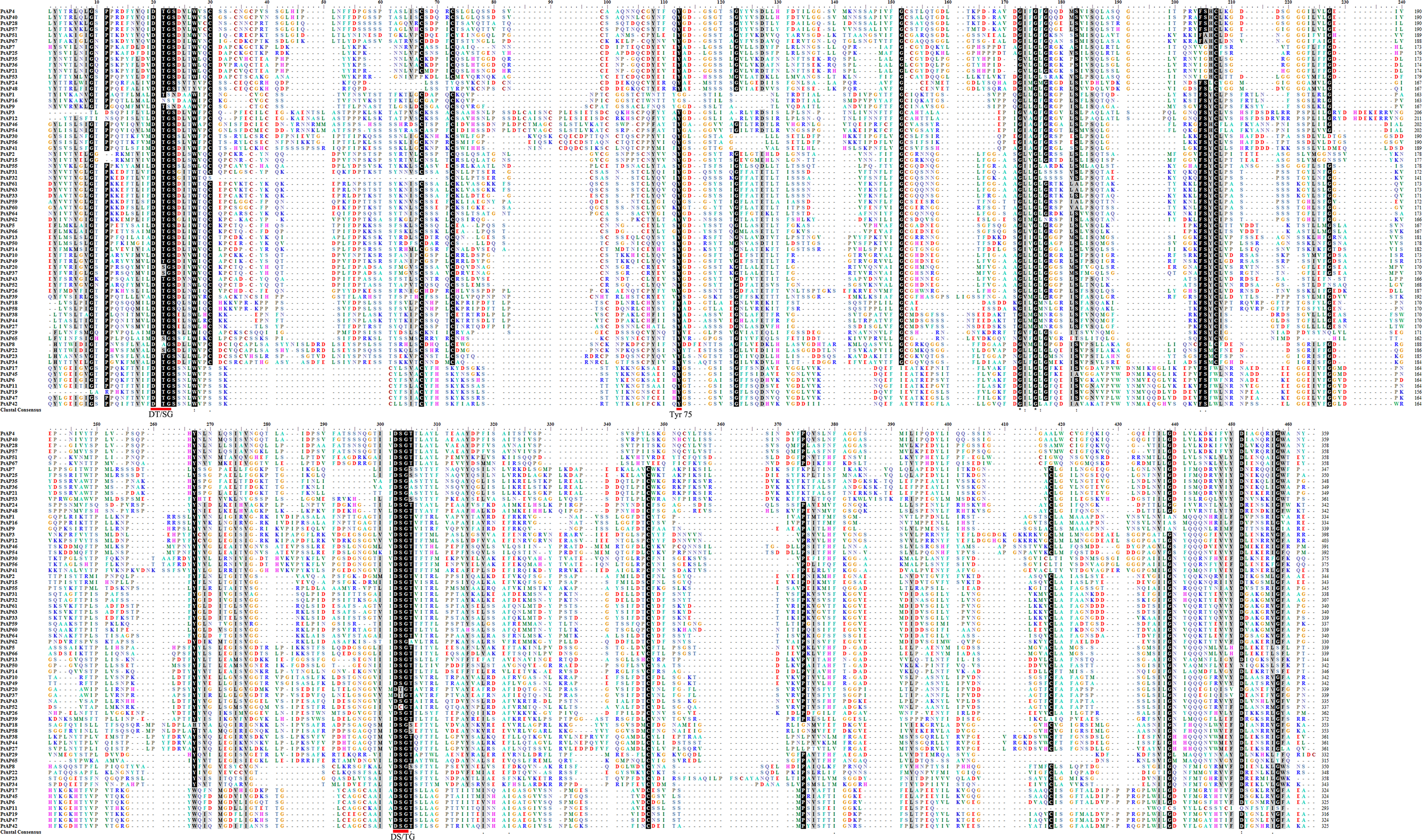

Supplement: Supplementary file 2 — Figure S1. Multiple sequence alignment of ASP domains in 67 PtAPs. The alignment was generated using Clustal X program with manual modification. The two conserved catalytic regions (DT/SG) of ASP domains and the invariant Tyr75 (pepsin numbering) are indicated. Identical and conserved residues are shaded in black and gray, respectively. (JPG 13825 kb) [file 12870_2019_1865_MOESM2_ESM.jpg]

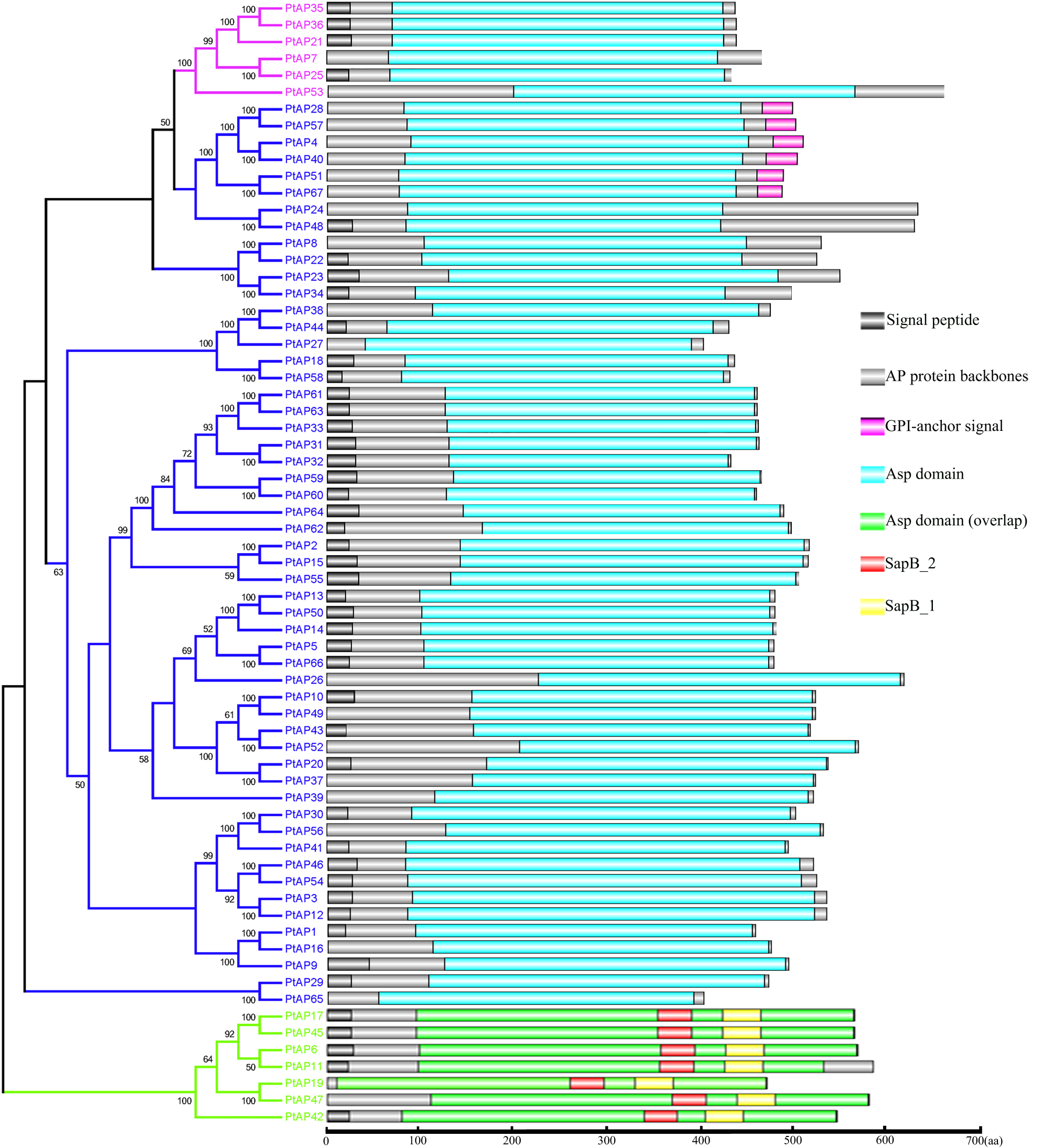

Supplement: Supplementary file 6 — Figure S2. Signal peptides and conserved domains of 67 PtAPs. Phylogenetic analysis of 67 PtAPs in the left. The distribution of signal peptides and conserved domains of PtAPs. (JPG 1762 kb) [file 12870_2019_1865_MOESM6_ESM.jpg]

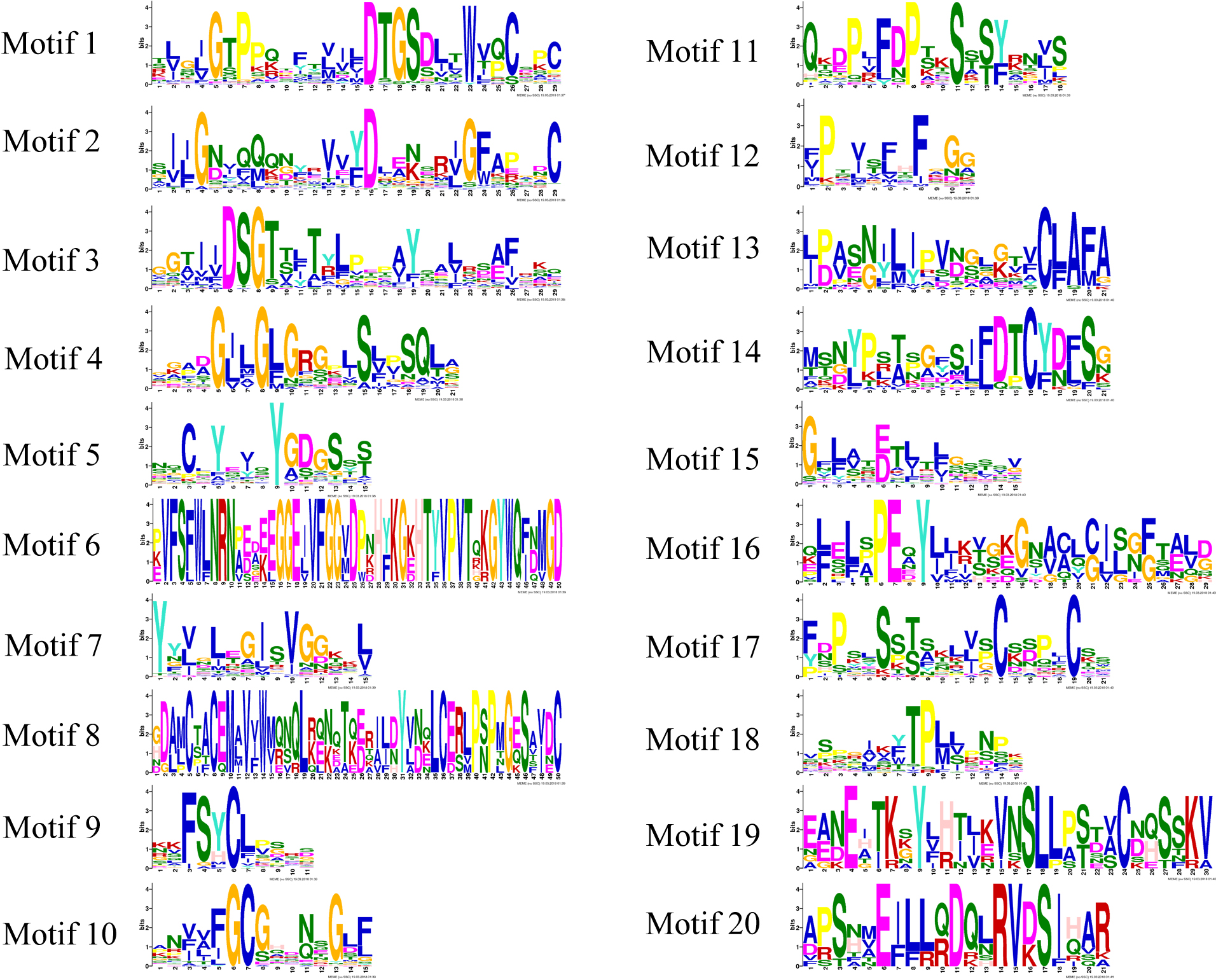

Supplement: Supplementary file 8 — Figure S3. Conserved motifs (1–20) identified by MEME online tool in 67 PtAPs. (JPG 1685 kb) [file 12870_2019_1865_MOESM8_ESM.jpg]
