# Supplementary material for: Genome-wide characterization of aspartic protease (AP) gene family in Populus trichocarpa and identification of the potential PtAPs involved in wood formation
Source: BMC Plant Biol. 2019 Jun 24;19:276. doi: 10.1186/s12870-019-1865-0 (PMC6591973; doi:10.1186/s12870-019-1865-0)
Supplement: Supplementary file 7 — Table S5. Backbones of 67 PtAP protein precursors. (DOCX 41 kb) [file 12870_2019_1865_MOESM7_ESM.docx]

Table S5. Backbones of 67 PtAP protein precursors

>PtAP1

MKAYLFSLAFLFLSLVQGLNTRGQGTTVKVFHVYSPQSPFRPSKPVSWEDSVLQMLAEDQARLQFLSSLVGRKSWVPIASGRQIVQSPTYIVKANVGTPAQTFLMALDTSNDAAWIPCNGCVGCSSTVFNSVTSTTFKTLGCDAPQCKQVPNPTCGGSTCTWNTTYGGSTILSNLTRDTIALSTDIVPGYTFGCIQKTTGSSVPPQGLLGLGRGPLSFLSQTQDLYKSTFSYCLPSFRTLNFSGTLRLGPAGQPLRIKTTPLLKNPRRSSLYYVNLIGIRVGRKIVDIPASALAFNPTTGAGTIFDSGTVFTRLVAPVYTAVRDEFRKRVGNAIVSSLGGFDTCYTGPIVAPTMTFMFSGMNVTLPPDNLLIRSTAGSTSCLAMAAAPDNVNSVLNVIANMQQQNHRILFDVPNSRIGVAREPCS*

>PtAP2

MVQAKPPFPSKLLLLSLLVSTLAIFDNGVQCFQGKKVLSMHKFQWKQGSNSSTCLSQETRWENGATILEMKHKDSCSGKILDWNKKLKKHLIMDDFQLRSLQSRMKSIISGRNIDDSVDAPIPLTSGIRLQTLNYIVTVELGGRKMTVIVDTGSDLSWVQCQPCKRCYNQQDPVFNPSTSPSYRTVLCSSPTCQSLQSATGNLGVCGSNPPSCNYVVNYGDGSYTRGELGTEHLDLGNSTAVNNFIFGCGRNNQGLFGGASGLVGLGRSSLSLISQTSAMFGGVFSYCLPITETEASGSLVMGGNSSVYKNTTPISYTRMIPNPQLPFYFLNLTGITVGSVAVQAPSFGKDGMMIDSGTVITRLPPSIYQALKDEFVKQFSGFPSAPAFMILDTCFNLSGYQEVEIPNIKMHFEGNAELNVDVTGVFYFVKTDASQVCLAIASLSYENEVGIIGNYQQKNQRVIYDTKGSMLGFAAEACTFD*

>PtAP3

MATSYSLLLCFSLCFSHFFISTSQTLFLPLTHSLSKTQFTSTHHLIKSTSTSSITRFRRHHHQKNTHNHRQVSLPLSPGSDYTLSFTLDSQPIFLYLDTGSDLVWFPCQPFECILCEGKAENTSLASTPPPKLSKTATPVSCKSSACSAAHSNLPSSDLCAISNCPLESIETSDCQKHSCPQFYYAYGDGSLIARLYRDSISLPLSNPTNLIVNNFTFGCAHTALAEPIGVAGFGRGVLSLPAQLATLSPQLGNQFSYCLVSHSFDSDRLRRPSPLILGRYDHDEKERRVNGVNKPRFVYTSMLDNLEHPYFYCVGLEGISIGRKKIPAPGFLRKVDGEGSGGLVVDSGTTFTMLPASLYGSVVAEFENRVGRVNERARVIEEDTGLSPCYYFDNNVVNVPSVVLHFVGNGSSVVLPRRNYFYEFLDGGDGKGKKRKVGCLMLMNGGDEAELSGGPGATLGNYQQQGFEVVYDLENKRVGFARRQCASLWETLNRD*

>PtAP4

MGPLFPAGILIAVVVFHATVVLSSFPATLHLERGVPASHKLKLSQLKERDRVRHSRMLQSSGGGVVDFPVQGTFDPFLVGFYFGSFCRLYYTRLQLGSPPRDFYVQIDTGSDVLWVSCSSCNGCPVSSGLHIPLNFFDPGSSPTASLISCSDQRCSLGLQSSDSVCAAQNNQCGYTFQYGDGSGTSGYYVSDLLHFDTILGGSVMKNSSAPIVFGCSTLQTGDLTKPDRAVDGIFGFGQQDMSVISQLASQGITPRVFSHCLKGDDSGGGILVLGEIVEPNIVYTPLVPSQPHYNLNLQSIYVNGQTLAIDPSVFATSSNQGTIIDSGTTLAYLTEAAYDPFISAITSTVSPSVSPYLSKGNQCYLTSSSINDVFPQVSLNFAGGTSMILIPQDYLIQQSSINGAALWCVGFQKIQGQEITILGDLVLKDKIFVYDIAGQRIGWANYDCSMSVNVSTAMNTGKSEYVNPGTLINNGSPENMPHKLIPVTMIPFLLHVLLLSCYLFL*

>PtAP5

MASMTSLCFVLALAMFTIFFSPAFSTSRRALEHPKMQKGFRVRLKHVDSGKNLTKLERIRHGVKRGRNRLQRLQAMALVASSSSEIEAPVLPGNGEFLMKLAIGTPPETYSAILDTGSDLIWTQCKPCTQCFHQSTPIFDPKKSSSFSKLSCSSQLCEALPQSSCNNGCEYLYSYGDYSSTQGILASETLTFGKASVPHVAFGCGADNEGSGFSQGAGLVGLGRGPLSLVSQLKEPKFSYCLTTVDDTKTSTLLMGSLASVNASSSAIKTTPLIHSPAHPSFYYLSLEGISVGDTRLPIKKSTFSLQDDGSGGLIIDSGTTITYLEESAFNLVAKEFTAKINLPVDSSGSTGLDVCFTLPSGSTNIEVPKLVFHFDGADLELPAENYMIGDSSMGVACLAMGSSSGMSIFGNVQQQNMLVLHDLEKETLSFLPTQCDLL*

>PtAP6

MGTILKPVAAATLFLCFLLLPMISSALSPPNDGLIRIGLKKRKYERNNRLAAKLESKEGESIKKYHLLRNLGGDAEDTDIVSLKNYMDAQYFGEIGIGTPPQKFTVIFDTGSSNLWVPSSKCYFSVACYFHSKYKSSHSRTYKENGKSAEIHYGTGAISGFFSQDHVKVGDLVVKNQEFIEATREPSVTFLVAKFDGILGLGFQEISVGKAVPVWYNMVEQGLVKEPVFSFWFNRNADEKEGGEIVFGGVDPDHYKGEHTYVPVTQKGYWQFDMGDVLIGGQTSGFCASGCAAIADSGTSLLAGPTTIITEVNHAIGATGVVSQECKAVVAQYGDTIMEMLLAKDQPQKICAQIGLCTFDGTRGVSMGIESVVNEHAQKASDGFHDAMCSTCEMAVVWMQNQLKQNQTQERILDYVNELCERLPSPMGESAVDCDGLSSMPNVSFTIGGRVFELSPEQYVLKVGEGDVAQCISGFTALDVPPPRGPLWILGDVFMGSFHTVFDYGNMRVGFAEAT*

>PtAP7

MEKKRKRIVSLVTMTLLFFIVMAANFRGCFSAASQTPIKGKSTTPANDRVGSSVFFRVTGNVYPTGHYSVILNIGNPPKAFDLDIDTGSDLTWVQCDAPCKGCTKPLDKLYKPKNNRVPCASSLCQAIQNNNCDIPTEQCDYEVEYADLGSSLGVLLSDYFPLRLNNGSLLQPRIAFGCGYDQKYLGPHSPPDTAGILGLGRGKASILSQLRTLGITQNVVGHCFSRVTGGFLFFGDHLLPPSGITWTPMLRSSSDTLYSSGPAELLFGGKPTGIKGLQLIFDSGSSYTYFNAQVYQSILNLVRKDLSGMPLKDAPEEKALAVCWKTAKPIKSILDIKSFFKPLTINFIKAKNVQLQLAPEDYLIITKDGNVCLGILNGGEQGLGNLNVIGDIFMQDRVVVYDNERQQIGWFPTNCNRLPNVDREYNEGFSQPYAAYFGILEEHCPATYASEKTKLFTKK*

>PtAP8

MECDRVVVLVVVVVLLCCQFEASIGLTFSSKLIHRFSDEAKSISISRKGNASGDLWPKRYSFEYFQLLLGNDLKRQRMKLGSQKNQLLFPSQGSQALFFGNELDWLHYTWIDIGTPNVSFLVALDAGSDLLWVPCDCIQCAPLSASYYNISLDRDLSEYSPSLSSTSRHLSCDHQLCEWGSNCKNPKDPCPYIFNYDDFENTTSAGFLVEDKLHLASVGDHTARKMLQASVVLGCGRKQGGSFFDGAAPDGVMGLGPGDISVPSLLAKAGLIQNCFSLCFDENDSGRILFGDRGHASQQSTPFLPIQGTYVAYFVGVESYCVGNSCLKRSGFKALVDSGSSFTYLPSEVYNELVSEFDKQVNAKRISFQDGLWDYCYNASSQELHDIPAIQLKFPRNQNFVVHNPTYSIPHHQGFTMFCLSLQPTDGSYGIIGQNFMIGYRMVFDIENLKLGWSNSSCQDTSDSADVHLAPPPDNKSPNPLPTNEQQSIPRTPSVAPAVAGRTSSESSAASLVIPFLHLMISLLLLLVKCMFISTL*

>PtAP9

MHTQQQSYLVRCSFSSESIAMDFPHCAATFFLFALLFSTTKAVDPCATQSDTSDLSVIPIYSKCSPFVPPKQESWVNTVITMASKDPERLKYLSTLADQKTTAVPIAPGQQVLKIANYVVRVKLGTPGQQMFMVLDTSNDAAWVPCSGCTGCSSTTFLPNASTTLGSLDCSGAQCSQVRGFSCPATGSSACLFNQSYGGDSSLTATLVQDAITLANDVIPGFTFGCINAVSGGSIPPQGLLGLGRGPISLISQAGAMYSGVFSYCLPSFKSYYFSGSLKLGPVGQPKSIRTTPLLRNPHRPSLYYVNLTGVSVGRIKVPIPSEQLVFDPNTGAGTIIDSGTVITRFVQPVYFAIRDEFRKQVNGPISSLGAFDTCFAATNEAEAPAITLHFEGLNLVLPMENSLIHSSSGSLACLSMAAAPNNVNSVLNVIANLQQQNLRIMFDTTNSRLGIARELCN*

>PtAP10

MEGKARNAPALLFFSFTCVFLSLSTTTLSTSPQFQTLTVNPLPNKPTLSWADTGPESEPETQTLTDSTSTEASTTTSLSVQLHHLDALSSDETPQDLFNSRLARDASRVKSLTSLAAAVGSTNRTRARGPGFSSSVTSGLAQGSGEYFTRLGVGTPARYVFMVLDTGSDVVWIQCAPCKKCYSQTDPVFNPTKSRSFANIPCGSPLCRRLDSPGCSTKKHICLYQVSYGDGSFTYGEFSTETLTFRGTRVGRVALGCGHDNEGLFIGAAGLLGLGRGRLSFPSQIGRRFSRKFSYCLVDRSASSKPSYMVFGDSAISRTARFTPLVSNPKLDTFYYVELLGVSVGGTRVPGITASLFKLDSTGNGGVIIDSGTSVTRLTRPAYVALRDAFRVGASNLKRAPEFSLFDTCFDLSGKTEVKVPTVVLHFRGADVSLPASNYLIPVDNSGSFCFAFAGTMSGLSIVGNIQQQGFRVVYDLAASRVGFAPRGCA*

>PtAP11

MGTQFTGIWIALLLSFPVVLSARDDGLMRIGLKKKKLDHLGRRVVPGSVNFIPKEEGGGASKPAATKKYYNIGETEADIVALKNYLDAQYYGEITIGTPPQTFTVIFDTGSSNLWVPSSKCYFSLACYFHSKYKSSASTTYVKNGTSAAIQYGTGSISGFFSQDSVEVGDLVVKNQGFIEATKEPGVTFLASKFDGILGLGFQEISVGNAVPVWYNMVNQGLVKEKVFSFWLNRNVEGEEGGEIVFGGVDPNHYKGEHTYVPVTHKGYWQFDMGDLLIGTETTGLCAGGCKAIADSGTSLLAGPTTVITQINNAIGASGIVSEECKTVVAQYGKIILEMLVAQAQPRKVCSQISFCTFDGTQGVSMNIESVVEENSDKSSDGLHDAMCTACEMMVVWMENRLRLNDTEDQILDYVNNLCDRLPSPNGESAVECSSLSSMPSISFEIGGKLFELSPEQVWQFCSVYLLCSSYCIQNFSYFISFNLIKLEGSRLIARVDLSINSILLHSSPLKCRSIYLVEIFFHVLFHIQI*

>PtAP12

MATSHSLLLCFILCFTHIFISTSQTLFLPLIHSLSKTQFTSTHHLLKSTSTRSTTRFHHHHHNKNSHNHRQVSLPLSPGSDYTLSFTINSQPISLYLDTGSDLVWFPCQPFECILCEGKAENASLASTPPPKLSKTATPVSCKSSACSAVHSNLPSSDLCAISNCPLESIEISDCRKHSCPQFYYAYGDGSLIARLYRDSIRLPLSNQTNLIFNNFTFGCAHTTLAEPIGVAGFGRGVLSLPAQLATLSPQLGNQFSYCLVSHSFDSDRVRRPSPLILGRYDHDEKERRVNGVKKPSFVYTSMLDNPRHPYFYCVGLEGISIGRKKIPAPDFLRKVDRKGSGGVVVDSGTTFTMLPASLYDFVVAEFENRVGRVNERASVIEENTGLSPCYYFDNNVVNVPRVVLHFVGNGSSVVLPRRNYFYEFLDGGHGKGKKRKVGCLMLMNGGDEAELSGGPGATLGNYQQQGFEVVYDLENRRVGFARRQCASLWEALNQN*

>PtAP13

MATFQSVLSFASAIALCVASFGCIYAHNAGFTTELVHRDSPKSPLYNSQQTHLQRWNKAMRRSVSRVHHFQRTAATVSPKEVESEIIANGGEYLMSLSLGTPPFQILAIADTGSDLIWTQCTPCDKCYKQIAPLFDPKSSKTYRDLSCDTRQCQNLGESSSCSSEQLCQYSYYYGDRSFTNGNLAVDTVTLPSTNGVPVYFPKTVIGCGRRNNGTFDKKDSGIIGLGGGPMSLISQMGSSIGGKFSYCLVPFSSESAGNSSKLHFGRNAVVSGSGVQSTPLISKNPDTFYYLTLEAMSVGDKKIEFGGSSFGGSEGNIIIDSGTSLTLFPVNFFTEFATAVENAVINGERTQDASGLLSHCYRPTPDLKVPVITAHFNGADVVLQTLNTFILISDDVLCLAFNSTQSGAIFGNVAQMNFLIGYDIQGKSVSFKPTDCTQL*

>PtAP14

MATTSFGFVTIVICFISLSPFPLLGAAASPDPGFSLNLIHRDSPLSPLYNPNHTDFDRLRNAFSRSISRVNVFKTKAVDINSFQNDLVPNGGEYFMKMSIGTPLVEVIVIADTGSDLTWVQCLPCDPCYRQKSPLFDPSRSSSYRHMLCGSRFCNALDVSEQACTMDTNICEYHYSYGDKSYTNGNLATEKFTIGSTSSRPVHLSPIVFGCGTGNGGTFDELGSGIVGLGGGALSLVSQLSSIIKGKFSYCLVPLSEQSNVTSKIKFGTDSVISGPQVVSTPLVSKQPDTYYYVTLEAISVGNKRLPYTNGLLNGNVEKGNVIIDSGTTLTFLDSEFFTELERVLEETVKAERVSDPRGLFSVCFRSAGDIDLPVIAVHFTDADVKLQPLNTFVKADEDLLCFTMISSNQIGIFGNLAQMDFLVGYDLEKRTVSFEPTDCTKH*

>PtAP15

MVQEKPAFPSKLLHLSLLVFLLTILDNGVQCFRGKKVLSMHKFQWKQESDSSSCLSQKSRWENGAVILEMKHKDSCSGKILDWNKKLQKRLIMDNFQLRSLQSRIKNIILSGNIDDSVDTQIPLTSGIRLQSLNYIVTVELGGRKMTVIVDTGSDLSWVQCQPCNRCYNQQDPVFNPSKSPSYRTVLCNSLTCRSLQLATGNSGVCGSNPPTCNYVVNYGDGSYTSGEVGMEHLNLGNTTVNNFIFGCGRKNQGLFGGASGLVGLGRTDLSLISQISPMFGGVFSYCLPTTEAEASGSLVMGGNSSVYKNTTPISYTRMIHNPLLPFYFLNLTGITVGGVEVQAPSFGKDRMIIDSGTVISRLPPSIYQALKAEFVKQFSGYPSAPSFMILDSCFNLSGYQEVKIPDIKMYFEGSAELNVDVTGVFYSVKTDASQVCLAIASLPYEDEVGIIGNYQQKNQRIIYDTKGSMLGFAEEACSFY*

>PtAP16

MTSQSLNTQSSFSFATMKAYLFSLAFLFCSLAQGLSTKGLGTTVKVFHVYSSQSPFRPSKPISWEESVLQMMAKDQARLQFLSSLVAKKSVVPIASGRGVIQSPSYIVKAKVGTPPQTLLMALDNSYDAAWIPCKGCVGCSSTVFNTVKSTTFKTLGCGAPQCKQVPNPICGGSTCTWNTTYGSSTILSNLTRDTIALSMDPVPYYAFGCIQKATGSSVPPQGLLGFGRGPLSFLSQTQNLYKSTFSYCLPSFRTLNFSGSLRLGPVGQPPRIKTTPLLKNPRRSSLYYVKLNGIRVGRKIVDIPRSALAFNPTTGAGTIFDSGTVFTRLVAPAYIAVRNEFRKRVGNATVSSLGGFDTCYSVPIVPPTITFMFSGMNVTMPPENLLIHSTAGVTSCLAMAAAPDNVNSVLNVIASMQQQNHRILFDVPNSRLGVAREQCS*

>PtAP17

MGVNLKAIVGFVFLSFLLFAVVSSASNDGLLRIGLKKVKLDKNNRIAARLDSKETLRASIRKYNLCGNLGESEDTDIVALKNYLDSQYYGEIGVGSPPQKFTVIFDTGSSNLWVPSSKCYLSVACYFHSKYDSGKSSTYKKNGKSAEIRYGSGSISGFFSNDAVEVGGLVVKDQEFIEATKEPNITFLVAKFDGILGLGFKEISVGDAVPVWDNMIKHGLIKEPVFSFWLNRNAEDEEGGEIVFGGMDPNHYKGKHTFVPVTRKGYWQFNMGDVHIGDKPTGYCASGCAAIADSGTSLLAGPTTIITMINQAIGASGVVSQQCKAVVSQYGEAIMDLLLSQAQPKRICSQIGLCTFDGTRGISIGIQSVVDEGNDKSSGVLGDAMCPACEMAVVWMRSQLKQNQTQDRILDYVNQLCERMPNPMGESAVDCESVPSMPTVAFTIGGKEFELAPEEYILKVGQGSAAQCISGFTALDIPPPRGPLWILGDIFMGRYHTVFDSGKLRVGFAEAA*

>PtAP18

MASPPLFKETMLPLLLLFTSLFLSTQETELKNDSLSFSFPLTSLPRSPQTSPSFYSSFISQSKKTPALKSAASPYNYRSRFKYSMILLVSLPIGTPPQSQQMILDTGSQLSWIQCHKKVPRKPPPSTVFDPSLSSSFSVLPCNHPLCKPRIPDFTLPTSCDLNRLCHYSYFYADGTLAEGNLVREKITFSTSQSTPPLILGCAEDASDDKGILGMNLGRLSFASQAKITKFSYCVPTRQVRPGFTPTGSFYLGENPNSAGFQYISLLTFSQSQRMPNLDPLAHTVALQGIRIGNKKLNIPVSAFRADPSGAGQSMIDSGSEFTYLVDVAYNKVREEVVRLAGPRLKKGYVYSGVSDMCFDGNAMEIGRLIGNMVFEFDKGVEIVIEKGRVLADVGGGVHCVGIGRSEMLGAASNIIGNFHQQNLWVEFDIANRRVGFGKADCSRSV*

>PtAP19

MLNTWEKLVLARPHKTSVIFDTGSSNLWVPSSKCYFSIACYFHSKYKSSLSSTYIKNGNSCEIHYGSGSISGFLSQDNVQVGGLVVKDQVFIEVTKEGSLSFVLGKFDGILGLGFQEISVGNVVPVWYNMVQQDLVDDEVFSFWLNRNPEAKEGGELVFGGVDPKHFKGKHTYVPVTKKGYWQINMGDFLIGNHSTGLCEEGCAAIVDSGTSLLAGPTPIITEINHAIGAEGVVSAECKEVVSQYGDLIWELLISGVKPNKVCAQLGLCIFNGDEYVSTGIESVVEKENKEGSSAGDDLLCIACEMLVIWVQNQLREKETKEAAINYLDKLCESLPSPMGESVIDCNSISTMPNISFTIGDKPFSLTPEQYVLKTGEGIAQVCISGFMALDVPPPRGPLWILGDVFMGVYHTVFDYGNLEVGFAEAA*

>PtAP20

MKNMSASRALLLLVVAMLLHLVTCTTTTTTTTSTATTHHNTITKAKTKTSSYPDFQLLNVKQAITETKIRPLKPSLYQELSMNIPNDSGSEGKQKLKLVHRDKVSFNNKFHNHSHVFHARMQRDVKRVVSLIRRVSSGSTASYGVEDFGSEVVSGMDQGSGEYFVRIGVGSPPRSQYMVIDSGSDIVWVQCKPCTQCYHQTDPLFDPADSASFMGVSCSSAVCDQVDNAGCNSGRCRYEVSYGDGSSTKGTLALETLTLGRTVVQNVAIGCGHMNQGMFVGAAGLLGLGGGSMSFVGQLSRERGNAFSYCLVSRVTNSNGFLEFGSEAMPVGAAWIPLIRNPHSPSYYYIGLSGLGVGDMKVPISEDIFELTELGNGGVVMDTGTAVTRFPTVAYEAFRDAFIDQTGNLPRASGVSIFDTCYNLFGFLSVRVPTVSFYFSGGPILTLPANNFLIPVDDAGTFCFAFAPSPSGLSILGNIQQEGIQISVDGANEFVGFGPNVC*

>PtAP21

MGKEKDGFCVVGVLVLFLFLGSSVTSDDSPKRWRKAMISGETMASSMLINRVPSSIVLPLHGNVYPNGYYNVTLNIGQPSKPYFLDVDTGSDLTWLQCDAPCVQCTEAPHPYYRPRNNLVPCMDPICQSLHSNGDHRCENPGQCDYEVEYADGGSSFGVLVRDTFNLNFTSEKRHSPLLALGLCGYDQFPGGSHHPIDGVLGLGKGKSSIVSQLSSLGLVRNVIGHCLSGHGGGFLFFGDDLYDSSRVAWTPMSPDAKHYSPGLAELTFDGKTTGFKNLLTTFDSGASYTYLNSQAYQGLISLLKKELSGKPLREALDDQTLPLCWKGRKPFKSIRDVKKYFKTFALSFTNERKSKTELEFPPEAYLIISSKGNACLGILNGTEVGLNDLNVIGDISMQDRVVIYDNEKERIGWAPGNCNRLPKSKSFII*

>PtAP22

MDAPFLLLILVMSSFLVQNTVELATFSSRLIHRFSKEYKEVSVSRGGDVNGTWWPEKKSKEYYQILVSSDLNRQKLKLGPHYQLLFPSQGSKTMSLGNDFGWLHYTWIDIGTPHVSFMVALDSGSDLFWVPCDCVQCAPLSASHYSSLDRDLSEYSPSQSSTSKQLSCSHRLCDMGPNCKNPKQSCPYSINYYTESTSSSGLLVEDIIHLASGGDDTLNTSVKAPVIIGCGMKQSGGYLDGVAPDGLLGLGLQEISVPSFLAKAGLIQNSFSMCFNEDDSGRIFFGDQGPATQQSAPFLKLNGNYTTYIVGVEVCCVGTSCLKQSSFSALVDSGTSFTFLPDDVFEMIAEEFDTQVNASRSSFEGYSWKYCYKTSSQDLPKIPSLRLIFPQNNSFMVQNPVFMIYGIQGVIGFCLAIQPADGDIGTIGQNFMMGYRVVFDRENLKLGWSRSNCGDEGDGKTLPLTPSGTPQNPLPTNEQQSTPGGHAVSPAVAVNAPSKPSAASSQLISTRFCLLKCLLPLYLLHRVVSAS*

>PtAP23

MATLFSYAHSFHVRHHKFPFLFLLFLFVLIERCSGFGTFGFDIHHRFSDPVKGMFGVDGLLPVKDSVPYFQVMAQRDRVIHGRRLATSTGGDNKNNKTLLTFYYGNETYRIDGLGLRNSCVSLYSNGLFGYILHYANVSVGTPSVSFLVALDTGSNLLWLPCDCSSCVHSLRSPSGTVDLNIYSPNTSSTSEKVPCNSTLCSQTQRDRCPSDQSNCPYQVVYLSNGTSTTGYIVQDLLHLISDDSQSKAVDAKITFGCGKVQTGSFLTGGAPNGLFGLGMSNISVPSTLAHNGYTSGSFSMCFSPNGIGRISFGDKGSTGQGETSFNQGQPRSSLYNISITQTSIGGQASDLVYSAIFDSGTSFTYLNDPAYTLIAESFNKLVKETRRSSTQVPFDYCYDIRSFISAQILPFSCAYANQTELTIPAVTLVMSGGDYFNITDPIVVVQLADVSAVYCLGMIKSGDVNIIGQNFMTGHRIVFDRERMILGWKPSNCYDNMDTNTLAVSPNTAVPPATAVNPEAKQIPASSPPGGSHSPRSKPFNFTLMMTLALFFAIV*

>PtAP24

MARLIQLTILFIQFFYYYYITTTTHGVSSNGTTFLLDSSRSAMILPLFLSPPNPCTKFSSTRRLLQRSNANALPNAHMRLHDDLLINGYYTTRLWIGTPPQRFALIVDTGSSVTYVPCSSCEQCGRHQDPKFQPDLSSTYQSVKCNIDCNCDDEKQQCVYERQYAEMSTSSGVLGEDIISFGNLSALAPQRAVFGCENMETGDLYSQHADGIMGMGRGDLSIVDHLVDKGVINDSFSLCYGGMGIGGGAMVLGGISPPSNMVFSQSDPVRSPYYNIDLKEIHVAGKPLPLNPTVFDGKHGTILDSGTTYAYLPEAAFVSFKDAIMKELHSLKPIRGPDPNYNDICFSGAGSDISQLSSSFPAVEMVFGNGQKLLLSPENYLFRHSKVHGAYCLGIFQNGKDPTTLLGGIVVRNTLVLYDRENSKIGFWKTNCSELWERLNVDGAPPPAPSSSNGNNSNTEMPPSVAPSDQKHYGLPDEKKIGQITFEMMLNVNYSDLKLHISELAESIAQELGINSSQVYILNSMEKGNASYIEWAVVPSGSADCISNVTALSIIARVAEYHLHLPDTFGSYHLINWEIKASAKRTWWQQHFLLVVLASAVTFIFGLLALGIWFIWRHRQRALNPYKPVDAVVTEQELQPL*

>PtAP25

MEKKSKRSVSLMMMMFLFFIVISADLQGCFSAASQTPIKGESSTPANDRVGSSVFFRVTGNVYPTGYYSVILNIGNPPKAFDFDIDTGSDLTWVQCDAPCKGCTKPRDKLYKPKNNLVPCSNSLCQAVSTGENYHCDAPDDQCDYEIEYADLGSSIGVLLSDSFPLRLSNGTLLQPKMAFGCGYDQKHLGPHPPPDTAGILGLGRGKVSILSQLRTLGITQNVVGHCFSRARGGFLFFGDHPFPSSRITWTPMLRSSSDTLYSSGPAELLFGGKPTGIKGLQLIFDSGSSYTYFNAQVYQSILNLVRKDLAGKPLKDAPEKELAVCWKTAKPIKSILDIESYFKPLTISFMNAKNVQLQLAPEDYLIITKDGNVCLGILNGSEQQLGNFNVIGDIFMQDRVVIYDNEKQQIGWFPANCDRLPQS*

>PtAP26

MKEEAERKKPDKKKKTRSFIVLLLFLLFSGAFEAIAGIHDHGKNVKSNISTLAGIELPDHMSFNAVSSSTTNTGCNLDTSKKVKQSQTIVSKEDFDLLEDDDDDDEGGEEEKEAKQTVKLHLKHRSKDRKSEGKESFVESTNRDLARIQTLHTRIIEKKNQNDISRLKKDKERPEKQIKTVVATAASPESYGTGLSGQLMATLESGVTLGSGEYFMDVFIGTPPKHYSLILDTGSDLNWIQCVPCHDCFEQNGPYYDPKESSSFRNIGCHDPRCHLVSSPDPPLPCKAENQTCPYFYWYGDSSNTTGDFATETFTVNLTSPTGKSEFKRVENVMFGCGHWNRGLFHGASGLLGLGRGPLSFSSQLQSLYGHSFSYCLVDRNSDTNVSSKLIFGEDKDLLNHPELNFTTLVGGKENPVDTFYYVQIKSIMVGGEVLNIPESTWNMTSDGVGGTIVDSGTTLSYFTEPAYQIIKDAFVKKVKGYPIVQDFPILDPCYNVSGVEKIDLPDFGILFADGAVWNFPVENYFIRLDPEEVVCLAILGTPRSALSIIGNYQQQNFHVLYDTKKSRLGYAPMNCADV*

>PtAP27

MESQHCLSSSPKMLILPLKTQVIPSGSVPRSPNKPPFHHNVSLIVSLTVGTPPQNVSMVIDTGSELSWLHCNKTLSYPTTFDPTRSTSYQTIPCSSPTCTNRTQDFPIPASCDSNNLCHATLSYADASSSDGNLASDVFHIGSSDISGLVFGCMDSVFSSNSDEDSKSTGLMGMNRGSLSFVSQLGFPKFSYCISGTDFSGLLLLGESNLTWSVPLNYTPLIQISTPLPYFDRVAYTVQLEGIKVLDKLLPIPKSTFEPDHTGAGQTMVDSGTQFTFLLGPVYNALRSAFLNQTSSVLRVLEDPDFVFQGAMDLCYLVPLSQRVLPLLPTVTLVFRGAEMTVSGDRVLYRVPGELRGNDSVHCLSFGNSDLLGVEAYVIGHHHQQNVWMEFDLEKSRIGLAQVRCDLAGQRFGVAL*

>PtAP28

MRGLWPPFVVIILVSFMFVSAVYCASLLHLERAFPLNNHGLELHQLRARDRLRHARLLQGFVGGVVDFSVQGSSDPYLVGLYFTKVKLGSPPREFNVQIDTGSDVLWVCCNSCNNCPRTSGLGIQLNFFDSSSSSTAGQVRCSDPICTSAVQTTATQCSSQTDQCSYTFQYGDGSGTSGYYVSDTLYFDAILGQSLIDNSSALIVFGCSAYQSGDLTKTDKAVDGIFGFGQGELSVISQLSTRGITPRVFSHCLKGDGSGGGILVLGEILEPGIVYSPLVPSQPHYNLNLLSIAVNGQLLPIDPAAFATSNSQGTIVDSGTTLAYLVAEAYDPFVSAVNAIVSPSVTPITSKGNQCYLVSTSVSQMFPLASFNFAGGASMVLKPEDYLIPFGSSEGGSAMWCIGFQKVQGVTILGDLVLKDKIFVYDLVKQRIGWANYDCSLSVNVSVTSSKDFINAGQLSVSSSSRDIMLFELLPLTVMVFLMHILLLEFQFL*

>PtAP29

MDTVFVLVSSLPLIFSTHFALTIANNLEFSSIQPTRLVTKLIHRDSIVSPYYRSNDTVADRTERTMKASLARLSYLYAKIERDFDINDLWLNLHPSASEPLFLVNFSMGQPPVPQLAIMDTGSSLLWIQCAPCKSCSQQIIGPMFDPSISSTYDSLSCKNIICRYAPSGECDSSSQCVYNQTYVEGLPSVGVIATEQLIFGSSDEGRNAVNNVLFGCSHRNGNYKDRRFTGVFGLGSGITSVVNQMGSKFSYCIGNIADPDYSYNQLVLSEGVNMEGYSTPLDVVDGHYQVILEGISVGETRLVIDPSAFKRTEKQRRVIIDSGTAPTWLAENEYRALEREVRNLLDRFLTPFMRESFLCYKGKVGQDLVGFPAVTFHFAEGADLVVDTEMRQASVYGKDFKDFSVIGLMAQQYYNVAYDLNKHKLFFQRIDCELLDE*

>PtAP30

MASSRFLLLLSFLILISSSSSTPTPTRTPTSTITIPLSAPSSTKLIVSSKNPWGALNHLASLSLSRAHHIKSPKTKFSLLKTPLFPRSYGGYSISLNFGTPPQTTKFVMDTGSSLVWFPCTSRYLCSRCDFPNIEVTGIPTFIPKQSSSSNLIGCKNHKCSWLFGPKVQSKCQECDPTTQNCTQSCPPYVIQYGLGSTAGLLLSETLDFPHKKTIPGFLVGCSLFSIRQPEGIAGFGRSPESLPSQLGLKKFSYCLVSHAFDDTPASSDLVLDTGSGSDDTKTPGLSYTPFQKNPTAAFRDYYYVLLRNIVIGDTHVKVPYKFLVPGSDGNGGTIVDSGTTFTFMEKPVYELVAKEFEKQVAHYTVATEVQNQTGLRPCFNISGEKSVSVPEFIFHFKGGAKMALPLANYFSFVDSGVICLTIVSDNMSGSGIGGGPAIILGNYQQRNFHVEFDLKNERFGFKQQNCVS*

>PtAP31

MANLISSIKFTGFIYVFLLFLCPLCSLKKGYAVEANEHIKKYVHTLEVNSLLASDSCDQSSKVIDKASSLQVLHKYGPCMQVLNDRSHVEFLLQDQLRVDSIQARLSKISGHGIFEEMVTKLPAQSGIAIGTGNYVVTVGLGTPKEDFTLVFDTGSGITWTQCQPCLGSCYPQKEQKFDPTKSTSYNNVSCSSASCNLLPTSERGCSASNSTCLYQIIYGDQSYSQGFFATETLTISSSDVFTNFLFGCGQSNNGLFGQAAGLLGLSSSSVSLPSQTAEKYQKQFSYCLPSTPSSTGYLNFGGKVSQTAGFTPISPAFSSFYGIDIVGISVAGSQLPIDPSIFTTSGAIIDSGTVITRLPPTAYKALKEAFDEKMSNYPKTNGDELLDTCYDFSNYTTVSFPKVSVSFKGGVEVDIDASGILYLVNGVKMVCLAFAANKDDSEFGIFGNHQQKTYEVVYDGAKGMIGFAAGACS*

>PtAP32

MANLISSIKFTGFIYVFLLFLCPLCSLKKGYAVEANEHIKKYVHTLEVNSLLASDSCDQSSKVIDKASSLQVLHKYGPCMQVLNDRSHVEFLLQDQLRVDSIQARLSKNSGHGIFEEMVTKLPAPSGIAIGTGNYVVTVGLGTPKEDFTLVFDTGSGITWTQSSCNLLPTSERGCSASNSTCLYQVIYGDQSSSKGFFATETLTISSSAVFSNFLFGCGQSNNGLFGKAAGLLGLSSSSVSLPSQTAEKYQKQFSYCLPSSPSSTGYLNFGGKVSQTAGFTPISPAFSSFYGIDIVGISVGGSQLPIDPSIFTKSGAIIDSGTVITRLPPTAYKALKEAFDEKMSNYPKTNGDELLDTCYDFSNYTTVSFPKVSVSFKGGVEVDINASGILYLVNGLKMVCLAFAANKDDSKIGIFGNHQQKTYEVVYDGAKGMVGFAAGGCS*

>PtAP33

MVTPISSISLTFFVNAFLLLCYLNKGHAVGEDEITKGYLHIIKVKSLLPSTACNQTFKVSNSLSLEVVHRSGPCIQVLNQEKAANAPSNMEILLQDRHRVDSIHARLSSHGVFQEKQATLPVQSGASIGSGDYAVTVGLGTPKKEFTLIFDTGSDLTWTQCEPCAKTCYKQKEPRLDPTKSTSYKNISCSSAFCKLLDTEGGESCSSPTCLYQVQYGDGSYSIGFFATETLTLSSSNVFKNFLFGCGQQNSGLFRGAAGLLGLGRTKLSLPSQTAQKYKKLFSYCLPASSSSKGYLSFGGQVSKTVKFTPLSEDFKSTPFYGLDITELSVGGNKLSIDASIFSTSGTVIDSGTVITRLPSTAYSALSSAFQKLMTDYPSTDGYSIFDTCYDFSKNETIKIPKVGVSFKGGVEMDIDVSGILYPVNGLKKVCLAFAGNGDDVKAAIFGNTQQKTYQVVYDDAKGRVGFAPSGCN*

>PtAP34

MRSSSKLTFFFLLITIWVFSKTCKGRVFTFKMHHRFSDSFKNWSGLTRNWPEKGSFEYYAALAHRDQMLRGRRLSDADASLAFSDGNSTFRISSLGFLHYTTVELGTPGVKFMVALDTGSDLFWVPCDCSRCAPTHGASYASDFELSIYNPRESSTSKKVTCNNDMCAQRNRCLGTFSSCPYIVSYVSAQTSTSGILVKDVLHLTTEDGGREFVEAYVTFGCGQVQSGSFLDIAAPNGLFGLGMEKISVPSVLSREGLIADSFSMCFGHDGIGRISFGDKGSPDQEETPFNVNPAHPTYNVTVTQARVGTMLIDVEFTALFDSGTSFTYMVDPAYSRVSEKFHSLARDKRRPPDPRIPFEYCYDMSPDANASLVPSMSLTMKGGRHFTVYDPIIVISTQNEIVYCLAVVKSTELNIIGQNFMTGYRVVFDREKLVLGWKKFDCYDVEDYNNFPLQPHASMVPPAVAAGLGSNSSTGSEKEASNKSPSSIASTYCYSHTSVFTSLISVFLICLLL*

>PtAP35

MGNEKVGFWVVGVLVLVLILGSSAASDDRQQRWRKAMMSGETMGSSMLMNRVPSSIVLPLHGNVYPTGFYNVTLNIGQPSKPYFLDVDTGSDLTWLQCDAPCVHCTEAPHPYYKPSNNLVACKDPICQSLHTGGDQRCENPGQCDYEVEYADGGSSLGVLVKDAFNLNFTSEKRQSPLLALGCGYDQLPGGTYHPIDGVLGLGRGKPSIVSQLSGLGLVRNVIGHCLSGRGGGFLFFGDDLYDSSRVAWTPMSPNAKHYSPGFAELTFDGKTTGFINLIVAFDSGASYTYLNSQAYQGLISLIKRELSTKPLREALDDQTLPICWKGRKPFKSVRDVKKYFKTFALSFANDGKSKTQLEFPPEAYLIVSSKGNACLGVLNGTEVGLNDLNVIGDISMQDRVVIYDNEKQLIGWAPGNCDRLPKSRSIII*

>PtAP36

MGKEKVGLWVVGVLVLVLILGLSAASDDRQQRWRKAMMSGETMGSSMLMNRVPSSIVLPLHGNVYPTGFYNVTLNIGQPSKPYFLDVDTGSDLTWLQCDVPRAQCTEAPHPYYKPSNNLVACKDPICQSLHTGGDQRCENPGQCDYEVEYADGGSSLGVLVKDAFNLNFTSEKRQSPLLALGLCGYDQLPGGTYHPIDGVLGLGRGKPSIVSQLSGLGLVRNVIGHCLSGRGGGFLFFGDDLYDSSRVAWTPMSPNAKHYSPGFAELTFDGKTTGFKNLIVAFDSGASYTYLNSQVYQGLISLIKRELSTKPLREALDDQTLPICWKGRKPFKSVRDVKKYFKTFALSFANDGKSKTQLEFPPEAYLIVSSKGNACLGVLNGTEVGLNDLNVIGDISMQDRVVIYDNEKQLIGWAPRNCDRIPKSRSIII*

>PtAP37

MLLQLAISTTTTKTSTNTSTATTHHDNISKAKTKTTLYPDFQLLNVKQALTETKTRPIKPSQYQELFKTNPNDSESGGKQKLKLVHRDKVSFSNKLHNHSHVFHARMHRDVKRVASLIHRLSSGSAAKYEVEDFGSDVVSGMNQGSGEYFVRIGLGSPPRSQYMVIDSGSDIVWVQCKPCTQCYHQTDPLFDPADSASFMGVSCSSAVCDRVENAGCNSGRCRYEVSYGDGSYTKGTLALETLTFGRTVVRNVAIGCGHSNRGMFVGAAGLLGLGGGSMSFMGQLSGQTGNAFSYCLVSRGTNTNGFLEFGSEAMPVGAAWIPLVRNPRAPSFYYIRLLGLGVGDTRVPVSEDVFQLNELGSGGVVMDTGTAVTRFPTVAYEAFRNAFIEQTQNLPRASGVSIFDTCYNLFGFLSVRVPTVSFYFSGGPILTIPANNFLIPVDDAGTFCFAFAPSPSGLSILGNIQQEGIQISVDEANEFVGFGPNIC*

>PtAP38

MIFVSIPKKKVGMQERCERRTDVCLTKFSIISKRKQSPNQGKQYISKQNIMAFLQLLVQLFISFILLQSKHCLSSNQPPIVLALRTQKHRTPISTPRLFSTTSKTTDKLLFHHNVTLTVSLTAGTPLQNITMVLDTGSELSWLHCKKEPNFNSIFNPLASKTYTKIPCSSPTCETRTRDLPLPVSCDPAKLCHFIISYADASSVEGNLAFETFRVGSVTGPATVFGCMDSGFSSNSEEDAKTTGLMGMNRGSLSFVNQMGFRKFSYCISDRDSSGVLLLGEASFSWLKPLNYTPLVEMSTPLPYFDRVAYSVQLEGIRVSDKVLSLPKSVFVPDHTGAGQTMVDSGTQFTFLLGPVYSALKQEFLLQTKGVLRVLNEPRYVFQGAMDLCYLIEPTRAALPNLPVVNLMFRGAEMSVSGQRLLYRVPGEVRGKDSVWCFTFGNSDSLGIESFVIGHHQQQNVWMEYDLEKSRIGFAEVRCDLAGQRLGLDV*

>PtAP39

MVRSFKSKQRVCFPLSHGHNTHTELHSTMVSLSLLFHLLLLAFVDLSTSTTEYLKLPLLHKTPFPTPLQSLSSDLQRLSLLHHSHHRHQNHRRTSSKSPLMSGASSGSGQYFVSIRLGSPPQTLLLVADTGSDLTWVRCSACKTNCSIHPPGSTFLARHSTTFSPTHCFSSLCQLVPQPNPNPCNHTRLHSTCRYEYVYSDGSKTSGFFSKETTTLNTSSGREMKLKSIAFGCGFHASGPSLIGSSFNGASGVMGLGRGPISFASQLGRRFGRSFSYCLLDYTLSPPPTSYLMIGDVVSTKKDNKSMMSFTPLLINPEAPTFYYISIKGVFVDGVKLHIDPSVWSLDELGNGGTVIDSGTTLTFLTEPAYREILSAFKREVKLPSPTPGGASTRSGFDLCVNVTGVSRPRFPRLSLELGGESLYSPPPRNYFIDISEGIKCLAIQPVEAESGRFSVIGNLMQQGFLLEFDRGKSRLGFSRRGCAVS*

>PtAP40

MGAAPFPAGILIAAVVFHATVVLSSFPATFHLERGITANYKLKLSKLKERDRVRHGRMLQSSGVGVVDFPVQGTFDPFLVGLYYTRLQLGTPPRDFYVQIDTGSDVLWVSCGSCNGCPVNSGLHIPLNFFDPGSSPTASLISCSDQRCSLGLQSSDSVCSAQNNLCGYNFQYGDGSGTSGYYVSDLLHFDTVLGGSVMNNSSAPIVFGCSALQTGDLTKSDRAVDGIFGFGQQDMSVVSQLASQGISPRAFSHCLKGDDSGGGILVLGEIVEPNIVYTPLVPSQPHYNLNMQSISVNGQTLAIDPSVFGTSSSQGTIIDSGTTLAYLAEAAYDPFISAITSIVSPSVRPYLSKGNHCYLISSSINDIFPQVSLNFAGGASMILIPQDYLIQQSSIGGAALWCIGFQKIQGQGITILGDLVLKDKIFVYDIANQRIGWANYDCSMSVNVSTAIDTGKSEFVNAGTLSNNGSPKNMPHKLTPVTMMSFLLHMLLLSCYMFL*

>PtAP41

MVSVSSSLLYCLSLLSLFPFISSSITIPLQHPQTNQIPFQDQYQKLNHLVTTSLARARHLKNPQTTPATTTTAPLFSHSYGGYSVSLSFGTPPQTLSFIMDTGSDIVWFPCTSHYLCKHCSFSSSSPSSRIQPFIPKESSSSKLLGCKNPKCSWIHHSNINCDQDCSIKSCLNQTCPPYMIFYGSGTTGGVALSETLHLHSLSKPNFLVGCSVFSSHQPAGIAGFGRGLSSLPSQLGLGKFSYCLLSHRFDDDTKKSSSLVLDMEQLDSDKKTNALVYTPFVKNPKVDNKSSFSVYYYLGLRRITVGGHHVKVPYKYLSPGEDGNGGVIIDSGTTFTFMAREAFEPLSDEFIRQIKDYRRVKEIEDAIGLRPCFNVSDAKTVSFPELRLYFKGGADVALPVENYFAFVGGEVACLTVVTDGVAGPERVGGPGMILGNFQMQNFYVEYDLRNERLGFKQEKCK*

>PtAP42

MESIRFLLISICMGAWLGGSLSSDGLARVGLKKRNLDLNSIHAARITRPQATSFARVTSNAEIVYLKNYLDTQYYGEIGIGSPPQIFTVVFDTGSSNLWVPSSKCLLSITCYFHSKFIARLSRTYTKIGIPCKIQYGSGSVSGFLSQDHVKVGDDIIINQEFAEVTREGFLALLGVQFDGILGLAFQDIAVAKATPVWYNMAEQGHVSQKVFSLWLNRNPSSELGGEVVFGGLDWRHFKGDHTYVPVTGRGYWQIQVGDIFIANNSTGLCAGGCSAIVDSGTSFLSGPTRIVAQINHAIGARGIVSLECKEVVSKYWNSIWDSMISGLRPEIICVDVGLCLYNNNTVIETVVDGEATDRLSVDEGGALCTFCEMIVFWIQVQLKEKKAKEKIFHYVDELCERLPNPLGKSFINCDEITAMPYVSFTIGNRSFPLSPEQYIVRVEESYATICLSGFAALDMPPRQGPLWILGDVFLGAYHTVFDFGNHRIGFAKAA*

>PtAP43

MGLLFYVFFSLFFASPPVSCSRILTPHPSETTVLDVAASIQRTKNIFSSGPKMSPFNQQEKETTSSELTVELLSRTSIQKTTHTGYKSLTLSRLQRDSARVKSLVTRLDLAINSISSSDLKPLETDSEFKPEDLQSPIISGTSQGSGEYFSRVGIGKPPSQAYLILDTGSDVNWVQCAPCADCYQQADPIFEPASSASFSTLSCNTRQCRSLDVSECRNDTCLYEVSYGDGSYTVGDFVTETITLGSAPVDNVAIGCGHNNEGLFVGAAGLLGLGGGSLSFPSQINATSFSYCLVDRDSESASTLEFNSTLPPNAVSAPLLRNHHLDTFYYVGLTGLSVGGELVSIPESAFQIDESGNGGVIVDSGTAITRLQTDVYNSLRDAFVKRTRDLPSTNGIALFDTCYDLSSKGNVEVPTVSFHFPDGKELPLPAKNYLVPLDSEGTFCFAFAPTASSLSIIGNVQQQGTRVVYDLVNHLVGFVPNKC*

>PtAP44

MVYLRLLVQLFISFIFLRSKQCFSSNQSPIILPLRIQNNHHISTRRLFSNSSSKTTGKLLFHHNVTLTASLTIGTPPQNITMVLDTGSELSWLRCKKEPNFTSIFNPLASKTYTKIPCSSQTCKTRTSDLTLPVTCDPAKLCHFIISYADASSVEGHLAFETFRFGSLTRPATVFGCMDSGSSSNTEEDAKTTGLMGMNRGSLSFVNQMGFRKFSYCISGLDSTGFLLLGEARYSWLKPLNYTPLVQISTPLPYFDRVAYSVQLEGIKVNNKVLPLPKSVFVPDHTGAGQTMVDSGTQFTFLLGPVYSALRKEFLLQTAGVLRVLNEPQYVFQGAMDLCYLIDSTSSTLPNLPVVKLMFRGAEMSVSGQRLLYRVPGEVRGKDSVWCFTFGNSDELGISSFLIGHHQQQNVWMEYDLENSRIGFAELRCDLAGQRLGLDVKNLA*

>PtAP45

MGVNLKAIGGFVLLSFLLFAVVLSESNDGLLRIGLKKVKFDKNNRIAARLDSQEALRASIRKYNLLGNLGESEDTDIVALKNYFDAQYYGEIGVGTPPQKFTVIFDTGSSNLWVPSSKCYLSVACYFHSKYNSGKSSSYKKNGKSAEIQYGSGSISGFFSIDAVEVGNLVVKDQEFIEATKEPSITFLVGKFDGILGLGFKEIAVGGAVPVWDNMIKQGLIKEPVFSFWLNRNADDEEGGEIVFGGMDPNHYKGKHTYVPVTQKGYWQFDMGDVIVGDKSTGYCAGGCAAIADSGTSLLAGPTAIITMINHAIGASGVVSQQCKAVVSQYGEVIMDLLLSEVQPKKICSQIGLCTFDGTRGISMGIQSVVDEGNDKSSGVLGDAMCSACEMAVFWMRSQLQQNQTQDRVLDYANQLCERVPNPTGQSTVDCGSVLSMPRIAFTIGGKEFELAPEEYILKVGQGSAAQCISGFTALDIPPPRGPLWILGDVFMGRYHTVFDSGKLRVGFAEAA*

>PtAP46

MAIMLNNITTFLFFLLVNSLLFYSIQSLARPRNPNSLILGLTPASRASLPTHPKASTSSRKKLTDVLDMMEPLREVRDGYLISLSIGTPPQVIQVYMDTGSDLTWAPCGNISFDCIECDNYRNNRMMASFSPSHSSSSHRDSCTSPFCIDVHSSDNPLDPCTMAGCSLSTLVKATCSWPCPPFAYTYGAGGVVAGTLTRDTLRVHGRNLGVTQEIPRFCFGCVASSYREPIGIAGFGRGALSLPSQLGFLRKGFSHCFLAFKYANNPNISSPLIIGDIALTSKDDMQFTPMLKSPMYPNYYYVGLEAITVGNVSATEVPSSLREFDSLGNGGMLVDSGTTYTHLPEPFYSQVLSVLQSIINYPRATDMEMRTGFDLCYKVPCQNNSILTGDLLPSITFHFLNNASLVLSRGSHFYAMSAPSNSTVVKCLLFQSMDDGDYGPAGVLGSFQQQDVEVVYDMEKERIGFRPMDCASAASFQGFNKT*

>PtAP47

MSALDFYTLFRLNSILHPRMGNKILLKAFCLWALTCFLLPASSNGLVRIGLKKRHLDLQTIKDARIARQEGKAGVGASSRVHDLGSSDGDIIPLKNYLDAQYLGEIGIGSPPQNFTVVFDTGSSNLWVPSSKCYFSIACYFHSKYKSSRSSTYTKNGNFCEIHYGSGSVSGFFSQDNVQVGDLVVKDQVFVEATKEGSLSFILGKFDGILGLGFQEISVGNVVPLWYNMIQQDLVDDEVFSFWLNRNPEAKEGGELVFGGVDPKHFKGKHTYVPVTQKGYWQINMGDFLIGKHSTGLCEGGCAAIVDSGTSLLAGPTPIITEINHAIGAEGLVSAECKEVVSHYGDLIWELIISGVQPSKVCTQLGLCIFNEAKSARTGIESVVEKENKEKSSAGNDLPCTACQMLVIWVQNQLREKATKETAINYLDKLCESLPSPMGQSSIDCNSISTMPNITFTIGDKPFSLTPEQYILKTGEGIAQVCISGFMALDVPPPRGPLWILGDVFMGAYHTIFDYGNLEVGFAEAA*

>PtAP48

MAYSSSSSSSIMISYSLILLNLYAIVSSTSDFNNRHHPTILPLLLSTPNISAHRMPFDGHYSRRHLQNSELPNARMRLFDDLLSNGYYTTRLFIGTPPQEFALIVDTGSTVTYVPCSSCEQCGKHQDPRFQPDLSSTYRPVKCNPSCNCDDEGKQCTYERRYAEMSSSSGVIAEDVVSFGNESELKPQRAVFGCENVETGDLYSQRADGIMGLGRGRLSVVDQLVDKGVIGDSFSLCYGGMDVGGGAMVLGQISPPPNMVFSHSNPYRSPYYNIELKELHVAGKPLKLKPKVFDEKHGTVLDSGTTYAYFPEAAFHALKDAIMKEIHHLKQIPGPDPNYHDICFSGAGREVSHLSKVFPEVNMVFGSGQKLSLSPENYLFRHTKVSGAYCLGIFQNGNDLTTLLGGIVVRNTLVTYDRENDKIGFWKTNCSELWKSLQVPGVPASAPVLSPSSNRSQEMPPAQAPSSMPFFHPGEIRIGIISFDMLISANNSNTKPNFTEVAEFIAHELEVDNLQVHMLNFTSTGNNYLVKWAILPAESADYISNTTAMKIIQQLSEHRLHFPERFGSYELVKWKFEPQKNRTWWQQHFVAVTVGVVVTLVVSLLSIGLWLVWRRQKALGTYVPVGAVGPEQELQPL*

>PtAP49

MEGKTRNASTLFFSFTCIFLFLSTTTTLSTSLQFQTLTLNPLPNKPTISWADTEPGTQTFTDQTTSEPSSSATTFLSVQLHHIDALSSDKSSQDLFNSRLVRDAARVKSLISLAATVGGTNLTRARGPGFSSSVISGLAQGSGEYFTRLGVGTPARYVYMVLDTGSDIVWIQCAPCIKCYSQTDPVFDPTKSRSFANIPCGSPLCRRLDYPGCSTKKQICLYQVSYGDGSFTVGEFSTETLTFRGTRVGRVVLGCGHDNEGLFVGAAGLLGLGRGRLSFPSQIGRRFNSKFSYCLGDRSASSRPSSIVFGDSAISRTTRFTPLLSNPKLDTFYYVELLGISVGGTRVSGISASLFKLDSTGNGGVIIDSGTSVTRLTRAAYVALRDAFLVGASNLKRAPEFSLFDTCFDLSGKTEVKVPTVVLHFRGADVPLPASNYLIPVDNSGSFCFAFAGTASGLSIIGNIQQQGFRVVYDLATSRVGFAPRGCA*

>PtAP50

MAAFRSPLSFALAIALLCVSGFGCIYARKVGFTVDLIHRDSPLSPFYNSEETDLQRINNALRRSISRVHHFDPIAAASVSPKAAESDVTSNRGEYLMSLSLGTPPFKIMGIADTGSDLIWTQCKPCERCYKQVDPLFDPKSSKTYRDFSCDARQCSLLDQSTCSGNICQYQYSYGDRSYTMGNVASDTITLDSTTGSPVSFPKTVIGCGHENDGTFSDKGSGIVGLGAGPLSLISQMGSSVGGKFSYCLVPLSSRAGNSSKLNFGSNAVVSGPGVQSTPLLSSETMSSFYFLTLEAMSVGNERIKFGDSSLGTGEGNIIIDSGTTLTIVPDDFFSNLSTAVGNQVEGRRAEDPSGFLSVCYSATSDLKVPAITAHFTGADVKLKPINTFVQVSDDVVCLAFASTTSGISIYGNVAQMNFLVEYNIQGKSLSFKPTDCTKK*

>PtAP51

MLMMMAEAQSRVLLLTMMISFTIVSANNGVFSVKYKYAGLQRSLSDLKAHDDQRQLRILAGVDLPLGGIGRPDILGLYYAKIGIGTPTKDYYVQVDTGSDIMWVNCIQCRECPKTSSLGIDLTLYNINESDTGKLVPCDQEFCYEINGGQLPGCTANMSCPYLEIYGDGSSTAGYFVKDVVQYARVSGDLKTTAANGSVIFGCGARQSGDLGSSNEEALDGILGFGKSNSSMISQLAVTGKVKKIFAHCLDGTNGGGIFVIGHVVQPKVNMTPLIPNQPHYNVNMTAVQVGHEFLSLPTDVFEAGDRKGAIIDSGTTLAYLPEMVYKPLVSKIISQQPDLKVHTVRDEYTCFQYSDSLDDGFPNVTFHFENSVILKVYPHEYLFPFEGLWCIGWQNSGVQSRDRRNMTLLGDLVLSNKLVLYDLENQAIGWTEYNCSSSIQVQDERTGTVHLVGYHYINSARSLNVQWAMLLLLLLSTLLLSLVC*

>PtAP52

MARGYTCCQPWLLVSKFASETSASTLNPPNSATKMSEPASKAFLLFLTLIFLFFTSTLPRDLPDATTTTTTTILDVASSLQQAHNILSFDLQTQKSSTHTTITTSTPSFSNSSLSFSLELHPRETIYKIHHKDYKSLVLSRLHRDTVRFNSLTARLQLALEDISKSDLKPLETEIKPEDLSTPVTSGTSQGSGEYFTRVGVGNPARQFYMVLDTGSDINWLQCQPCTDCYQQTDPIFDPTASSTYAPVTCQSQQCSSLEMSSCRSGQCLYQVNYGDGSYTFGDFATESVSFGNSGSVKNVALGCGHDNEGLFVGAAGLLGLGGGPLSLTNQLKATSFSYCLVNRDSAGSSTLDFNSAQLGVDSVTAPLMKNRKIDTFYYVGLSGMSVGGQMVSIPESTFRLDESGNGGIIVDCGTAITRLQTQAYNPLRDAFVRMTQNLKLTSAVALFDTCYDLSGQASVRVPTVSFHFADGKSWNLPAANYLIPVDSAGTYCFAFAPTTSSLSIIGNVQQQGTRVTFDLANNRMGFSPNKCQ*

>PtAP53

MESDDDQSPQLKGVVIISLPPPDNPSLGKTITAFTLTNNDYPQSHQTPQTHQEDQLPISSPPPPPSQNSQLQFPSSRLFLGTPRKLLSFVFISLFALAIYSSLFTNTFQELKSNNNDDDDQKPKSYVFPLYHKLGIREIPLNDLENHLRRFVYKENLVASVDHLNGPHKISKLASSNAAAAMDSSAIFPVRGNLYPDGLYFTYMLVGSPPQPYYLDFDTGSDLTWIQCDAPCTSCAKGANAWYKPRRGNIVPPKDLLCMEVQRNQKAGYCETCDQCDYEIEYADHSSSMGVLATDKLLLMVANGSLTKLNFIFGCAYDQQGLLLKTLVKTDGILGLSRAKVSLPSQLASQGIINNVIGHCLTTDLGGGGYMFLGDDFVPRWGMAWVPMLDSPSMEFYHTEVVKLNYGSSPLSLGGMESRVKHILFDSGSSYTYFPKEAYSELVASLNEVSGAGLVQSTSDTTLPLCWRANFPIRSVKDVKKFFKTLTFQFGTKWLVISTKFRIPPEGYLMMSDKGNVCLGILEGSKVHDGSTIILGDISLRGQLVVYDNVNKKIGWTPSDCAKPKRSDSLQFFDGLPFFDGTERHAEYCGHRGIGARPLRGGCVYGYMSLLTTQHNTRSIFFLVNLLTLKTIQECIWRDLRLLTCLGSLNVILYK*

>PtAP54

MAIALNKNITTFLFFLLVNSLVSYSIQSLASPRNPNSLILGLTLASRASFPTYPKASTSSRKIVSIDVLGAKKPSREVRDGYLISLNIGTPPQVIQVLMDTGSDLTWVPCGNLSFDCMECDDYRNNKLMATFSPSYSSSSYRASCASPFCIDIHSSDNPLDTCTVAGCSLSTLVKATCSRPCPSFAYTYGAGGVVTGILTRDTLRVNGSSPGVAKEIPKFCFGCVGSAYREPIGIAGFGRGTLSMVSQLGFLQKGFSHCFLAFKYANNPNISSPLVVGDIALTSKDDMQFTPMLNSPMYPNFYYVGLEAITVGNVSATEVPSSLREFDSLGNGGMKIDSGTTYTHLPEPFYSQVLSILQSTINYPRDTGMEMQTGFDLCYKVPRPNNNTLTSDDLLPSITFHFLNNVSLVLPQGNHFYPVSAPGNPAVVKCLMFQSTDDGDDGPAGVFGSFQQQNVEVVYDLEKERIGFQPMDCASAASSQGLHKT*

>PtAP55

MESISCKSLEAMMGLLWIMVCYLSLASSSSQAKPKAYVQSINQSSIHLNIYHVHGHGSSLTPNSSSLLSDVLLHDEEHVKALSDRLANKGLGSGSAKPPKSGHLLEPNSASIPLNPGLSIGSGNYYVKLGLGTPPKYYAMILDTGSSLSWLQCQPCAVYCHAQADPLYDPSVSKTYKKLSCASVECSRLKAATLNDPLCETDSNACLYTASYGDTSFSIGYLSQDLLTLTSSQTLPQFTYGCGQDNQGLFGRAAGIIGLARDKLSMLAQLSTKYGHAFSYCLPTANSGSSGGGFLSIGSISPTSYKFTPMLTDSKNPSLYFLRLTAITVSGRPLDLAAAMYRVPTLIDSGTVITRLPMSMYAALRQAFVKIMSTKYAKAPAYSILDTCFKGSLKSISAVPEIKMIFQGGADLTLRAPSILIEADKGITCLAFAGSSGTNQIAIIGNRQQQTYNIAYDVSTSRIGFAPGSCH*

>PtAP56

MAPFPSITKTFYFLIFTNSLTNHNQEEVEEEKEENNTFMASSLLLFLLSFLILITSSKSTTITIPLSAPSFNKLIVSSKKPWGSLNHLASLSLSRAHHIKSPKTNFSLIKTPLFPRSYGGYSISLNFGTPPQTTKFVMDTGSSLVWFPCTSRYLCSECNFPNIKKTGIPTFLPKLSSSSKLIGCKNPRCSMIFGPEIQSKCQECDSTAQNCTQTCPPYVIQYGSGSTAGLLLSETLDFPNKKTIPDFLVGCSIFSIKQPEGIAGFGRSPESLPSQLGLKKFSYCLVSHAFDDTPTSSDLVLDTGSGSGVTKTAGLSHTPFLKNPTTAFRDYYYVLLRNIVIGDTHVKVPYKFLVPGTDGNGGTIVDSGTTFTFMENPVYELVAKEFEKQMAHYTVATEIQNLTGLRPCYNISGEKSLSVPDLIFQFKGGAKMALPLSNYFSIVDSGVICLTIVSDNVAGPGLGGGPAIILGNYQQRNFYVEFDLENEKFGFKQQSCA*

>PtAP57

MRGLWPPFFITILLLLLLLFMSVSVVYCASLLQLERAFPLNNHGLELSQLRARDRLRHARLLQGFVGGVVDFSVQGSPDPYLVGLYFTKVKLGSPPREFNVQIDTGSDVLWVCCNSCNNCPRTSGLGIQLNFFDSSSSSTAGLVHCSDPICTSAVQTTVTQCSPQTNQCSYTFQYEDGSGTSGYYVSDTLYFDAILGESLVVNSSALIVFGCSTFQSGDLTMTDKAVDGIFGFGQGELSVISQLSTHGITPRVFSHCLKGEGIGGGILVLGEILEPGMVYSPLVPSQPHYNLNLQSIAVNGKLLPIDPSVFATSNSQGTIVDSGTTLAYLVAEAYDPFVSAVNVIVSPSVTPIISKGNQCYLVSTSVSQMFPLASFNFAGGASMVLKPEDYLIPFGPSQGGSVMWCIGFQKVQGVTILGDLVLKDKIFVYDLVRQRIGWANYDCSLSVNVSVTSSKDFINAGQLSVSSSSRDIMLFELLPLTVMVLTMHILLLEFKFL*

>PtAP58

MLLFYLFLLLTSCSLSAQETQHKNDSLSFSFPLTSLPRSPQASPNFYPSFISQTKKASTLKSSSFSSSPYNYRSGFKYSMILLVSLPIGTPPQTQQMILDTGSQLSWIQCHKKVPRKPPPSSVFDPSLSSSFSVLPCNHPLCKPRIPDFTLPTSCDQNRLCHYSYFYADGTLAEGNLVREKITFSRSQSTPPLILGCAEESSDAKGILGMNLGRLSFASQAKLTKFSYCVPTRQVRPGFTPTGSFYLGENPNSGGFRYINLLTFSQSQRMPNLDPLAYTVAMQGIRIGNQKLNIPISAFRPDPSGAGQTMIDSGSEFTYLVDEAYNKVREEVVRLVGARLKKGYVYGGVSDMCFNGNAIEIGRLIGNMVFEFDKGVEIVVEKERVLADVGGGVHCVGIGRSEMLGAASNIIGNFHQQNIWVEFDLANRRVGFGKADCSRSV*

>PtAP59

MASTPLSPMSLTFILYVFLVLLCPLCSLKKGLTVEGKETTKNYIRTVRVNSLLPSNVCSQSTRVLNRASSLKVVNKYGPCIPVTGAPKTINVPSTAEFLLQDQLRVKSFQVRLSMNPSSGVFKEMQTTIPASIVPTGGAYVVTVGLGTPKKDFTLSFDTGSDLTWTQCEPCLGGCFPQNQPKFDPTTSTSYKNVSCSSEFCKLIAEGNYPAQDCISNTCLYGIQYGSGYTIGFLATETLAIASSDVFKNFLFGCSEESRGTFNGTTGLLGLGRSPIALPSQTTNKYKNLFSYCLPASPSSTGHLSFGVEVSQAAKSTPISPKLKQLYGLNTVGISVRGRELPINGSISRTIIDSGTTFTFLPSPTYSALGSAFREMMANYTLTNGTSSFQPCYDFSNIGNGTLTIPGISIFFEGGVEVEIDVSGIMIPVNGLKEVCLAFADTGSDSDFAIFGNYQQKTYEVIYDVAKGMVGFAPKGC*

>PtAP60

MATPISLVFLLYVLLFLLCPLCSLKKGHTVAANEITKGYFRNVNVNSLLPSSVCDHSNKVLNKASSLKVVSKYGPCTVTGDPKTFPSAAEILRRDQLRVKSIRAKHSMNSSTTGVFNEMKTRVPTTHFGGGYAVTVGLGTPKKDFSLLFDTGSDLTWTQCEPCSGGCFPQNDEKFDPTKSTSYKNLSCSSEPCKSIGKESAQGCSSSNSCLYGVKYGTGYTVGFLATETLTITPSDVFENFVIGCGERNGGRFSGTAGLLGLGRSPVALPSQTSSTYKNLFSYCLPASSSSTGHLSFGGGVSQAAKFTPITSKIPELYGLDVSGISVGGRKLPIDPSVFRTAGTIIDSGTTLTYLPSTAHSALSSAFQEMMTNYTLTKGTSGLQPCYDFSKHANDNITIPQISIFFEGGVEVDIDDSGIFIAANGLEEVCLAFKDNGNDTDVAIFGNVQQKTYEVVYDVAKGMVGFAPGGC*

>PtAP61

MATPICSISLLSVFLLLFSLEKGYAVEENEATKSYLHIIKVNSLLPTTACNHSSKVSNSLSLEVVHRHGPCIGIVNQEKGADAPSNMEIFLRDQNRVDSIHARLSSRGMFPEKQATTLPVQSGASIGAGDYVVTVGLGTPKKEFTLIFDTGSDITWTQCEPCVKTCYKQKEPRLNPSTSTSYKNISCSSALCKLVASGKKFSQSCSSSTCLYQVQYGDGSYSIGFFATETLTLSSSNVFKNFLFGCGQQNNGLFGGAAGLLGLGRTKLALPSQTAKTYKKLFSYCLPASSSSKGYLSLGGQVSKSVKFTPLSADFDSTPFYGLDITGLSVGGRQLSIDESAFSAGTVIDSGTVITRLSPTAYSELSSAFQNLMTDYPSTSGYSIFDTCYDFSKYDTVRIPKVGVTFKGGVEMDIDVSGILYPVNGLKKVCLAFAGNDDDSDTSIFGNVQQRTYQVVYDGAKGRVGFAPGGCS*

>PtAP62

MRVRILCCCCCLMLLLQLLARHNNCLREEYLSRIGAPMSSKSLLSFVIYGFLLLSPCNSLKDNADEGTRAYFHTLKISSLPSTEVCKESSKALNEGSSSLKLVHRFGPCNPHRTSTAPASSFNEILRRDKLRVDSIIQARRSMNLTSSVEHMKSSVPFYGLSKITASDYIVNVGIGTPKKEMPLIFDTGSGLIWTQCKPCKACYPKVPVFDPTKSASFKGLPCSSKLCQSIRQGCSSPKCTYLTAYVDNSSSTGTLATETISFSHLKYDFKNILIGCSDQVSGESLGESGIMGLNRSPISLASQTANIYDKLFSYCIPSTPGSTGHLTFGGKVPNDVRFSPVSKTAPSSDYDIKMTGISVGGRKLLIDASAFKISSTIDSGAVLTRLPPKAYSALRSVFREMMKGYPLLDQDDFLDTCYDFSNYSTVAIPSISVFFEGGVEMDIDVSGIMWQVPGSKVYCLAFAELDDEVSIFGNFQQKTYTVVFDGAKERIGFAPGGCD*

>PtAP63

MATPICSISLLSVFLLLFSLEKGYAVEENEATKSYLHIIKVNSLLPTTACNHSSKVSNSLSLEVVHRHGPCIGIVNQEKGADAPSNMEIFLRDQNRVDSIHARLSSRGMFPEKQATTLPVQSGASIGAGDYVVTVGLGTPKKEFTLIFDTGSDITWTQCEPCVKTCYKQKEPRLNPSTSTSYKNISCSSALCKLVASGKKFSQSCSSSTCLYQVQYGDGSYSIGFFATETLTLSSSNVFKNFLFGCGQQNNGLFGGAAGLLGLGRTKLALPSQTAKTYKKLFSYCLPASSSSKGYLSLGGQVSKSVKFTPLSADFDSTPFYGLDITGLSVGGRKLSIDESAFSAGTVIDSGTVITRLSPTAYSELSSAFQNLMTDYPSTSGYSIFDTCYDFSKYDTVRIPKVGVTFKGGVEMDIDVSGILYPVNGLKKVCLAFAGNDDDSDTSIFGNVQQRTYQVVYDGAKGRVGFAPGGCS*

>PtAP64

MATPISSVSLTMRCFLYAYFLCLCLLFSLEKGYALEGRKVAESHHSHSIEVSSLLPSASCKPSTKVLSNNDNKASLKVVHKHGPCSKLSQDEASAAPTHTEILLQDQSRVKSIHSRLSNSKTSGGKDVKVTDSTTIPAKDGSTVGSGNYIVTVGLGTPKKDLSLIFDTGSDITWTQCQPCARSCYKQKEQIFDPSQSTSYTNISCSSSICNSLTSATGNTPGCASSACVYGIQYGDSSFSVGFFGTEKLTLTSTDAFNNIYFGCGQNNQGLFGGSAGLLGLGRDKLSVVSQTAQKYNKIFSYCLPSSSSSTGFLTFGGSASKNAKFTPLSTISAGPSFYGLDFTGISVGGKKLAISASVFSTAGAIIDSGTVITRLPPAAYSALRASFRNLMSKYPMTKALSILDTCYDFSSYTTISVPKIGFSFSSGIEVDIDATGILYASSISQVCLAFAGNSDATDVFIFGNVQQKTLEVFYDGSAGKVGFAPGGCS*

>PtAP65

MLKSFTCKPDPTGNKYCCCTDPRSSANVLSGLVSDSHGIRTTLLLSQPSELFYINFSIGHPPLPQLAIMDSGSSFLWIKCLPCSPCSSKSPISIFDPRKSLTYSSMSCRRYRCNHSKCNSYNECTYNTTYVRGPGSTGIYVFEQLSFETIDDTKIVVPRVLLGCGRNLEVDKGQYNGVFGLGVGRETSLITQLGSQFSYCVGNIMDPHILTTSYPWKAAMVAIYYVTLEGISIEGKLLEIDRRIFERTAMVDNGVILDSGTAYTWLAQDAYNALSEEVQSLFREMLQRYKGMPNQLCYIGSVREDLSGFPAVTFHFANGAQLVLDTQSSSAVNGDNSKNLSVIGMMAQQNYNVGYDIGQNKLYFQRIDCELLKD*

>PtAP66

MANMSSLSLVVALAIFAFVFSHAFSTSRRVLEHPKVQNGFRAKLKHVDSGKNLTKFERIQHGVKRGRHRLQRFKAMALVASSNSEIDAPVLPGNGEFLMKLAIGTPPETYSAIMDTGSDLIWTQCKPCTQCFDQPTPIFDPKKSSSFSKLSCSSKLCEALPQSTCSDGCEYLYGYGDYSSTQGMLASETLTFGKVSVPEVAFGCGEDNEGSGFSQGSGLVGLGRGPLSLVSQLKEPKFSYCLTSVDDTKASTLLMGSLASVKASDSEIKTTPLIQNSAQPSFYYLSLEGISVGDTSLPIKKSTFSLQEDGSGGLIIDSGTTITYLEQSAFDLVAKEFTSQINLPVDNSGSTGLEVCFTLPSGSTDIEVPKLVFHFDGADLELPAENYMIADASMGVACLAMGSSSGMSIFGNIQQQNMLVLHDLEKETLSFLPTQCDEL*

>PtAP67

MDLRRLVALLMVLLVVQLGLVSFSLGNVVFKVQHKFAGRLRNLTEFKAHDARRHSRLLSSVDLPLGGNGHPAETGLYFAKIGLGNPSKDYYVQVDTGSDILWVNCIGCDKCPTKSDLGIKLTLYDPASSVSATRVSCDDDFCTSTYNGLLPDCKKELPCQYNVVYGDGSSTAGYFVSDAVQFERVTGNLQTGLSNGTVTFGCGAQQSGGLGTSGEALDGILGFGQANSSMISQLASAGKVKRAFAHCLDNVNGGGIFAIGELVSPKVNTTPMVPNQAHYNVYMKEIEVGGTVLELPTDVFDSGDRRGTIIDSGTTLAYLPEVVYDSMMNEIRSQQPGLSLHTVEEQFICFKYSGNVDDGFPDIKFHFKDSLTLTVYPHDYLFQISEDIWCFGWQNGGMQSKDGRDMTLLGDLVLSNKLVLYDIENQAIGWTEYNCSSSIKLKDDKSGSVYSVGAENLSSASPVLSGRIVTFLLLLFAMLHRFT*

Green: Signal peptide; Blue: ASP domain; Pink: GPI-anchor signal;

Red: SapB_2 [region](app:ds:region)s; Yellow: SapB_1 [region](app:ds:region)s; Asp domain (overlap)
