# Supplementary material for: Genome-wide characterization of aspartic protease (AP) gene family in Populus trichocarpa and identification of the potential PtAPs involved in wood formation
Source: BMC Plant Biol. 2019 Jun 24;19:276. doi: 10.1186/s12870-019-1865-0 (PMC6591973; doi:10.1186/s12870-019-1865-0)
Supplement: Supplementary file 9 — Table S6. Estimated divergence period of PtAP gene pairs in Populus trichocarpa. (DOCX 15 kb) [file 12870_2019_1865_MOESM9_ESM.docx]

| **Gene pairs** | **Ka** | **Ks** | **Ka/Ks** | **MYA** |
| --- | --- | --- | --- | --- |
| *PtAP1* *& PtAP16* | 0.0716 | 0.2012 | 0.3559 | 11.05 |
| *PtAP2 & PtAP15* | 0.0699 | 0.2344 | 0.2982 | 12.88 |
| *PtAP3 & PtAP12* | 0.0419 | 0.2692 | 0.1556 | 14.79 |
| *PtAP4 & PtAP40* | 0.0479 | 0.2393 | 0.2002 | 13.15 |
| *PtAP5 & PtAP66* | 0.0636 | 0.3416 | 0.1862 | 18.77 |
| *PtAP7 & PtAP25* | 0.0745 | 0.2790 | 0.2670 | 15.33 |
| *PtAP8 & PtAP22* | 0.3289 | 1.6215 | 0.2028 | 89.09 |
| *PtAP10 & PtAP49* | 0.0557 | 0.3759 | 0.1482 | 20.65 |
| *PtAP17 & PtAP45* | 0.0479 | 0.2417 | 0.1982 | 13.28 |
| *PtAP18 & PtAP58* | 0.0561 | 0.4636 | 0.1210 | 25.47 |
| *PtAP19 & PtAP47* | 0.0705 | 0.1899 | 0.3712 | 10.43 |
| *PtAP20 & PtAP37* | 0.0774 | 0.3799 | 0.2037 | 20.87 |
| *PtAP21 & PtAP35* | 0.0655 | 0.2938 | 0.2229 | 16.14 |
| *PtAP23 & PtAP34* | 0.4875 | 2.0764 | 0.2348 | 114.09 |
| *PtAP24 & PtAP48* | 0.2834 | 1.2433 | 0.2279 | 68.31 |
| *PtAP28 & PtAP57* | 0.0391 | 0.1723 | 0.2269 | 9.47 |
| *PtAP30 & PtAP56* | 0.0659 | 0.2434 | 0.2707 | 13.37 |
| *PtAP38 & PtAP44* | 0.0724 | 0.3740 | 0.1936 | 20.55 |
| *PtAP43 & PtAP52* | 0.3010 | 2.0027 | 0.1503 | 110.04 |
| *PtAP46 & PtAP54* | 0.0890 | 0.2488 | 0.3577 | 13.67 |
| Notes: Ka, non-synonymous substitution rate; Ks, synonymous substitution rate; MYA, million years ago | | | | |

Table S6. Estimated divergence period of PtAP gene pairs in *Populus trichocarpa*
