# Supplementary material for: Genome-wide characterization of aspartic protease (AP) gene family in Populus trichocarpa and identification of the potential PtAPs involved in wood formation
Source: BMC Plant Biol. 2019 Jun 24;19:276. doi: 10.1186/s12870-019-1865-0 (PMC6591973; doi:10.1186/s12870-019-1865-0)
Supplement: Supplementary file 10 — Table S7. Probes of PtAP genes in Populus trichocarpa. (DOCX 32 kb) [file 12870_2019_1865_MOESM10_ESM.docx]

Table S7. Probes of *PtAP* genes in *Populus trichocarpa*

| Gene symbol | Group | Gene Locus | Probe set |
| --- | --- | --- | --- |
| *PtAP1* | C | Potri.001G028200 | PtpAffx.4070.1.A1_s_at |
| *PtAP2* | C | Potri.001G041700 | None |
| *PtAP3* | C | Potri.001G158600 | PtpAffx.215609.1.S1_s_at |
| *PtAP4* | C | Potri.001G213600 | [Ptp.1422.1.S1_s_at](https://www.affymetrix.com/analysis/netaffx/fullrecord.affx?pk=POPLAR:PTP.1422.1.S1_S_AT) |
| *PtAP5* | C | Potri.001G306200 | None |
| *PtAP6* | A | Potri.001G356900 | [Ptp.4619.1.S1_at](https://www.affymetrix.com/analysis/netaffx/fullrecord.affx?pk=POPLAR:PTP.4619.1.S1_AT) |
| *PtAP7* | B | Potri.002G081400 | [PtpAffx.140528.1.A1_a_at](https://www.affymetrix.com/analysis/netaffx/fullrecord.affx?pk=POPLAR:PTPAFFX.140528.1.A1_A_AT) |
| *PtAP8* | C | Potri.002G092100 | [PtpAffx.22769.1.A1_at](https://www.affymetrix.com/analysis/netaffx/fullrecord.affx?pk=POPLAR:PTPAFFX.22769.1.A1_AT) |
| *PtAP9* | C | Potri.002G104600 | Ptp.2098.2.S1_at |
| *PtAP10* | C | Potri.002G171700 | Ptp.7531.1.S1_at |
| *PtAP11* | A | Potri.002G228300 | Ptp.2368.1.S1_s_at |
| *PtAP12* | C | Potri.003G076300 | [PtpAffx.203018.1.S1_at](https://www.affymetrix.com/analysis/netaffx/fullrecord.affx?pk=POPLAR:PTPAFFX.203018.1.S1_AT) |
| *PtAP13* | C | Potri.003G087900 | [PtpAffx.215128.1.S1_at](https://www.affymetrix.com/analysis/netaffx/fullrecord.affx?pk=POPLAR:PTPAFFX.215128.1.S1_AT) |
| *PtAP14* | C | Potri.003G105300 | [PtpAffx.203136.1.S1_at](https://www.affymetrix.com/analysis/netaffx/fullrecord.affx?pk=POPLAR:PTPAFFX.203136.1.S1_AT) |
| *PtAP15* | C | Potri.003G185100 | None |
| *PtAP16* | C | Potri.003G195500 | None |
| *PtAP17* | A | Potri.004G007600 | [Ptp.1829.1.S1_s_at](https://www.affymetrix.com/analysis/netaffx/fullrecord.affx?pk=POPLAR:PTP.1829.1.S1_S_AT) |
| *PtAP18* | C | Potri.004G085000 | PtpAffx.318.1.A1_at |
| *PtAP19* | A | Potri.005G002800 | [PtpAffx.31479.1.S1_a_at](https://www.affymetrix.com/analysis/netaffx/fullrecord.affx?pk=POPLAR:PTPAFFX.31479.1.S1_A_AT) |
| *PtAP20* | C | Potri.005G063000 | [Ptp.2693.1.A1_at](https://www.affymetrix.com/analysis/netaffx/fullrecord.affx?pk=POPLAR:PTP.2693.1.A1_AT) |
| *PtAP21* | B | Potri.005G069600 | [PtpAffx.152850.1.A1_a_at](https://www.affymetrix.com/analysis/netaffx/fullrecord.affx?pk=POPLAR:PTPAFFX.152850.1.A1_A_AT) |
| *PtAP22* | C | Potri.005G079900 | [PtpAffx.2733.1.A1_at](https://www.affymetrix.com/analysis/netaffx/fullrecord.affx?pk=POPLAR:PTPAFFX.2733.1.A1_AT) |
| *PtAP23* | C | Potri.005G108700 | [PtpAffx.225666.1.S1_s_at](https://www.affymetrix.com/analysis/netaffx/fullrecord.affx?pk=POPLAR:PTPAFFX.225666.1.S1_S_AT) |
| *PtAP24* | C | Potri.005G144600 | Ptp.580.1.A1_at |
| *PtAP25* | B | Potri.005G179900 | None |
| *PtAP26* | C | Potri.005G204600 | PtpAffx.121010.1.A1_at |
| *PtAP27* | C | Potri.006G087600 | PtpAffx.206252.1.S1_s_at |
| *PtAP28* | C | Potri.006G118800 | None |
| *PtAP29* | C | Potri.006G179500 | [PtpAffx.206545.1.S1_at](https://www.affymetrix.com/analysis/netaffx/fullrecord.affx?pk=POPLAR:PTPAFFX.206545.1.S1_AT) |
| *PtAP30* | C | Potri.006G204700 | PtpAffx.1069.1.S1_at |
| *PtAP31* | C | Potri.006G232400 | [PtpAffx.206696.1.S1_s_at](https://www.affymetrix.com/analysis/netaffx/fullrecord.affx?pk=POPLAR:PTPAFFX.206696.1.S1_S_AT) |
| *PtAP32* | C | Potri.006G232500 | [PtpAffx.206696.1.S1_s_at](https://www.affymetrix.com/analysis/netaffx/fullrecord.affx?pk=POPLAR:PTPAFFX.206696.1.S1_S_AT) |
| *PtAP33* | C | Potri.006G232600 | [PtpAffx.165.1.S1_at](https://www.affymetrix.com/analysis/netaffx/fullrecord.affx?pk=POPLAR:PTPAFFX.165.1.S1_AT) |
| *PtAP34* | C | Potri.007G063800 | [PtpAffx.207249.1.S1_at](https://www.affymetrix.com/analysis/netaffx/fullrecord.affx?pk=POPLAR:PTPAFFX.207249.1.S1_AT) |
| *PtAP35* | B | Potri.007G099200 | [PtpAffx.1986.1.S1_at](https://www.affymetrix.com/analysis/netaffx/fullrecord.affx?pk=POPLAR:PTPAFFX.1986.1.S1_AT) |
| *PtAP36* | B | Potri.007G099300 | [PtpAffx.1986.1.S1_at](https://www.affymetrix.com/analysis/netaffx/fullrecord.affx?pk=POPLAR:PTPAFFX.1986.1.S1_AT) |
| *PtAP37* | C | Potri.007G106300 | PtpAffx.139136.1.S1_at |
| *PtAP38* | C | Potri.008G058000 | [PtpAffx.207715.1.S1_at](https://www.affymetrix.com/analysis/netaffx/fullrecord.affx?pk=POPLAR:PTPAFFX.207715.1.S1_AT) |
| *PtAP39* | C | Potri.008G115900 | [Ptp.7838.1.S1_s_at](https://www.affymetrix.com/analysis/netaffx/fullrecord.affx?pk=POPLAR:PTP.7838.1.S1_S_AT) |
| *PtAP40* | C | Potri.009G001700 | [Ptp.742.1.A1_at](https://www.affymetrix.com/analysis/netaffx/fullrecord.affx?pk=POPLAR:PTP.742.1.A1_AT) |
| *PtAP41* | C | Potri.009G162400 | None |
| *PtAP42* | A | Potri.010G003400 | [PtpAffx.208435.1.S1_at](https://www.affymetrix.com/analysis/netaffx/fullrecord.affx?pk=POPLAR:PTPAFFX.208435.1.S1_AT) |
| *PtAP43* | C | Potri.010G128200 | [PtpAffx.148858.1.S1_at](https://www.affymetrix.com/analysis/netaffx/fullrecord.affx?pk=POPLAR:PTPAFFX.148858.1.S1_AT) |
| *PtAP44* | C | Potri.010G201400 | [PtpAffx.209330.1.S1_at](https://www.affymetrix.com/analysis/netaffx/fullrecord.affx?pk=POPLAR:PTPAFFX.209330.1.S1_AT) |
| *PtAP45* | A | Potri.011G007600 | [Ptp.1959.1.S1_s_at](https://www.affymetrix.com/analysis/netaffx/fullrecord.affx?pk=POPLAR:PTP.1959.1.S1_S_AT) |
| *PtAP46* | C | Potri.012G118000 | [PtpAffx.218364.1.S1_at](https://www.affymetrix.com/analysis/netaffx/fullrecord.affx?pk=POPLAR:PTPAFFX.218364.1.S1_AT) |
| *PtAP47* | A | Potri.013G002200 | PtpAffx.31479.1.S1_a_at |
| *PtAP48* | C | Potri.014G020200 | [PtpAffx.13718.1.S1_at](https://www.affymetrix.com/analysis/netaffx/fullrecord.affx?pk=POPLAR:PTPAFFX.13718.1.S1_AT) |
| *PtAP49* | C | Potri.014G099400 | [PtpAffx.2376.1.S1_at](https://www.affymetrix.com/analysis/netaffx/fullrecord.affx?pk=POPLAR:PTPAFFX.2376.1.S1_AT) |
| *PtAP50* | C | Potri.014G114400 | [PtpAffx.211686.1.S1_at](https://www.affymetrix.com/analysis/netaffx/fullrecord.affx?pk=POPLAR:PTPAFFX.211686.1.S1_AT) |
| *PtAP51* | C | Potri.014G146400 | [PtpAffx.211835.1.S1_s_at](https://www.affymetrix.com/analysis/netaffx/fullrecord.affx?pk=POPLAR:PTPAFFX.211835.1.S1_S_AT) |
| *PtAP52* | C | Potri.015G051800 | [Ptp.5843.1.S1_s_at](https://www.affymetrix.com/analysis/netaffx/fullrecord.affx?pk=POPLAR:PTP.5843.1.S1_S_AT) |
| *PtAP53* | B | Potri.015G053300 | PtpAffx.18489.1.A1_s_at |
| *PtAP54* | C | Potri.015G113100 | [PtpAffx.212954.1.S1_at](https://www.affymetrix.com/analysis/netaffx/fullrecord.affx?pk=POPLAR:PTPAFFX.212954.1.S1_AT) |
| *PtAP55* | C | Potri.016G000600 | PtpAffx.11702.1.A1_at |
| *PtAP56* | C | Potri.016G071900 | None |
| *PtAP57* | C | Potri.016G096700 | [Ptp.7463.1.S1_at](https://www.affymetrix.com/analysis/netaffx/fullrecord.affx?pk=POPLAR:PTP.7463.1.S1_AT) |
| *PtAP58* | C | Potri.017G131800 | None |
| *PtAP59* | C | Potri.018G014500 | [Ptp.2249.1.A1_at](https://www.affymetrix.com/analysis/netaffx/fullrecord.affx?pk=POPLAR:PTP.2249.1.A1_AT) |
| *PtAP60* | C | Potri.018G014600 | [PtpAffx.125956.1.S1_at](https://www.affymetrix.com/analysis/netaffx/fullrecord.affx?pk=POPLAR:PTPAFFX.125956.1.S1_AT) |
| *PtAP61* | C | Potri.018G014700 | [PtpAffx.160855.1.S1_at](https://www.affymetrix.com/analysis/netaffx/fullrecord.affx?pk=POPLAR:PTPAFFX.160855.1.S1_AT) |
| *PtAP62* | C | Potri.018G014800 | Ptp.5519.1.S1_s_at |
| *PtAP63* | C | Potri.018G014900 | [PtpAffx.160855.1.S1_at](https://www.affymetrix.com/analysis/netaffx/fullrecord.affx?pk=POPLAR:PTPAFFX.160855.1.S1_AT) |
| *PtAP64* | C | Potri.018G015100 | [PtpAffx.653.8.S1_s_at](https://www.affymetrix.com/analysis/netaffx/fullrecord.affx?pk=POPLAR:PTPAFFX.653.8.S1_S_AT) |
| *PtAP65* | C | Potri.018G106500 | None |
| *PtAP66* | C | Potri.019G002100 | [Ptp.1623.1.S1_at](https://www.affymetrix.com/analysis/netaffx/fullrecord.affx?pk=POPLAR:PTP.1623.1.S1_AT) |
| *PtAP67* | C | Potri.019G054900 | PtpAffx.212288.1.S1_at |
